# Health conditions in adults with HIV compared with the general population: A population-based cross-sectional analysis

**DOI:** 10.1016/j.eclinm.2022.101392

**Published:** 2022-04-21

**Authors:** Daniel R. Morales, David Moreno-Martos, Nashaba Matin, Patricia McGettigan

**Affiliations:** a Division of Population Health and Genomics, University of Dundee, Dundee, United Kingdom; b Department of Public Health, University of Southern Denmark, Denmark; c Division of Population Health and Genomics, University of Dundee, United Kingdom; d Barts Health NHS Trust, Grahame Hayton Unit, Royal London Hospital, London, United Kingdom; e William Harvey Research Institute, Queen Mary University of London, United Kingdom

**Keywords:** HIV, Comorbidity, Multimorbidity

## Abstract

**Background:**

Life expectancy in adults with human immunodeficiency virus (HIV) has increased and managing other health conditions is increasingly important for patients and healthcare planning. The aim of this study was to examine the prevalence and association between different health conditions and HIV status.

**Methods:**

We performed a cross-sectional analysis of adult UK Clinical Practice Research Datalink primary care electronic medical records linked to hospital admissions as of Nov 30, 2015. We examined 47 health condition groups and 304 physical and mental health conditions by HIV status, after adjustment for age, sex, social deprivation status using logistic regression.

**Findings:**

There were 964 patients with HIV (61.7% male; 92.8% aged <65 years) and 941,113 non-HIV patients (49.4% male; 75.2% aged <65 years). Condition groups with the greatest prevalence in HIV that were also highly prevalent in adults without HIV included: lipid disorder (41.4% vs 40.2%), and hypertension (19.1% vs 24.6%). Following adjustment, 18 (37.5%) condition groups were more likely in adults with HIV and ten (20.8%) were less likely. Individual conditions that were less likely in adults with HIV included: atrial fibrillation (odds ratio [OR] 0.37 [95% CI 0.20–0.64]) and hypertension (OR_0.78 [0.65–0.94]); rheumatoid arthritis (OR 0.27 [0.05–0.84]); asthma (OR_0.65 (0.53–0.80]); and certain eye diseases such as macular degeneration (OR_0.30 [0.09–0.70]). Meanwhile individual conditions that were more likely included: liver fibrosis, sclerosis, and cirrhosis (OR_3.23 [1.85–5.20]); pulmonary embolism (OR_2.06 [1.15–3.36]); male infertility (OR_2.23 [1.50–3.16]) and female infertility (OR_2.01 [1.34–2.88]); bipolar disorder (OR_2.93 [1.52–5.05]) and depression (OR_1.49 [1.28–1.71]); cervical malignancy (OR_4.64 [1.15–12.15]); and infections.

**Interpretation:**

Comorbidity is common in adults with HIV, with physical and mental health conditions spanning a wide spectrum. HIV management should consider multidisciplinary care models to provide optimal patient care.

**Funding:**

The project was funded by the Bart’s Charity; DRM was funded by a Wellcome Trust Clinical Research Career Development Fellowship; DRM and DMM received funding from the HDR-UK Precision therapeutics programme.

**Research in context**

**Evidence before this study**

Life expectancy in adults with HIV has increased and managing comorbidity is increasingly important. We searched PubMed from inception to Oct 28, 2021, for observational studies measuring the prevalence of comorbidities amongst adults with HIV compared to the general population using the search terms (HIV AND (comorbidity) AND (prevalence) AND (cross-section*) AND (primary care)). We identified no large-scale evaluation of health conditions spanning the spectrum of physical and mental health conditions compared to the general population.

**Added value of this study**

This study comprehensively measures the prevalence of 47 health condition groups and 304 separate health conditions through a standardised approach using linked health data. The findings add to the evidence that comorbidities in adults with HIV are common and should be accounted for.

**Implications of all the available evidence**

This study accurately quantifies known comorbidities in adults with HIV and highlights associations with other health conditions. As life expectancy of people living with HIV in the UK approximates that of the general population, managing comorbidity should become an important feature for future healthcare planning from both specialist and primary care services.

Alt-text: Unlabelled box

## Introduction

It was estimated that 0.15% of the United Kingdom (UK) population was living with human immunodeficiency virus (HIV) in 2020, of whom, 4.8% were estimated to be unaware of their infection, a fall from 0.16% and 13%, respectively in 2015.^1^^,^^2^ Mortality in adults with HIV has also fallen since highly active antiretroviral therapy was first introduced in the UK and those who start treatment soon after diagnosis now have a life expectancy more closely approximating that of the general population.^3^, ^4^, ^5^ This means people with HIV will increasingly develop other health conditions, the burden of which will continue to increase as the HIV population ages. This will require co-management that can be complex to deliver in single disease speciality clinics. Despite comorbidity in the general population being common, many single disease guideline recommendations rely upon evidence where individuals with multimorbidity are excluded or are under-represented.^6^^,^^7^ For instance, several studies have reported an increased risk of cardiovascular disease in adults with HIV. This recognition has led to changes in some HIV care pathways such as the integration of joint cardiovascular for adults with HIV.^8^ Meanwhile, Canadian, Danish and Dutch studies have reported that people with HIV are also at higher risk of multiple other morbidities.^9^, ^10^, ^11^ There is also a lack of evidence on the effectiveness of interventions for other health conditions in people with HIV and a comprehensive understanding of physical and mental health comorbidity in these individuals would better inform future care pathways. However, the number of health conditions that have been evaluated to date have been limited and comparisons often rely upon controls sampled from the entire population. The aim of this study was therefore to measure the prevalence and association between a large number of physical and health conditions in adults with HIV compared to the general population.

## Methods

### Study population and data source

We conducted a cross-sectional analysis of anonymised patient data from the UK Clinical Practice Research Datalink (CPRD) GOLD, which contains de-identified electronic primary care records for use in public health research, capturing diagnoses, symptoms, prescriptions, referrals and tests.^12^ The data relate to around 7% of the UK population and are broadly representative of the population in terms of age, sex, and ethnicity with good validity.^13^^,^^14^ Additional data for deaths and index of multiple deprivation were derived through linkage with the UK Office of National Statistics (ONS). Data on hospitalisations was obtained via linkage to the Hospital Episodes Statistics (HES), which provide information on diagnoses and medical procedures related to all elective and emergency hospital admissions across all National Health Service (NHS) hospitals in England. Study approval was granted by the independent scientific advisory committee of the Medicines and Healthcare products Regulatory Agency (protocol 15_199RA2R).^15^ Informed consent was not a requirement for this study.

### Study population

The cross-section consisted of all acceptable patients aged ≥18 years registered for at least two years at general practices contributing up-to-standard linked data to CPRD GOLD as of the index date (November 30, 2015).

### HIV cases

People with HIV were defined based on a coded diagnosis or record of a positive HIV test in CPRD GOLD or coded diagnosis in HES at any point during the study period (Appendix:Table A1 and Appendix:Table A2).

### Health conditions

We examined the presence of 304 physical and mental health conditions that have been described elsewhere.^16^ Health conditions were measured individually and also after grouping related conditions into 47 higher-level health condition groups. Health conditions were defined using either Read codes recorded within the primary care or international classification of disease, tenth revision (ICD-10) codes recorded in HES.^17^ Algorithms defining these conditions were based on diagnosis or procedural codes recorded at any point prior to the index date. Chronic kidney disease (CKD) was additionally defined by the inclusion of blood test values with only the most recent blood test value prior to index date being used. Biochemically defined CKD was calculated as having an estimated glomerular filtration rate of less than 60 ml/min/ 1.73 m^2^. Lipid disorders were defined biochemically using the following cut-offs to define abnormal lipids: total cholesterol (TC) >5 mmol/l; low-density lipoprotein cholesterol (LDL-C) >3 mmol/l, triglycerides (TG) >2.3 mmol/l; and high-density lipoprotein cholesterol (HDL-C) <1 mmol/l. When a code or test was not recorded it was assumed that a person did not have the diagnosis. The proportion of patients with a lipid and eGFR blood test are presented in Appendix: Table A3.

### Statistical analysis

We calculated the prevalence of each health condition group and individual health condition amongst people with HIV and the non-HIV population. We used logistic regression to calculate crude and adjusted odds ratios (OR) for the probability of each health condition, or condition group (binary variable), in people with HIV compared to the non-HIV population adjusting for age (continuous variable), sex (binary variable) and the index of multiple deprivation (continuous variable).^18^ Lipids were modelled additionally including a categorical missing variable when no blood test was recorded.

### Role of the funding source

No funders had any role in study design; in the collection, analysis, and interpretation of data; in the writing of the report; and in the decision to submit the paper for publication. DMM and DRM had full access to the data and all authors made the decision to submit for publication.

## Results

Of 942,077 patients, 964 (0.1%) were identified with HIV. The characteristics of adults with and without HIV are shown in Table 1. Compared to those without HIV, a greater proportion of adults with HIV were men than women (61.7% vs 49.4%), were living in more deprived areas and were aged between 25 and 64 years (90.8% vs 66.5%), with few aged 65 years and over (7.2% vs 24.8%).

**Table 1:** Demographics of adults with and without HIV.

Category	People with HIV, n (%) (*N* = 964)	People without HIV, n (%) (*N* = 941,113)	Percentage difference (95% CI)
Sex			
Male	595 (61.7)	464,530 (49.4)	12.3 (9.1 to 15.4)
Female	369 (38.3)	476,576 (50.6)	−12.3 (−15.4 to −9.1)
Age group (years)			
18–24	19 (2)	81,338 (8.6)	−6.6 (−7.4 to −5.5)
25–44	383 (39.7)	291,124 (30.9)	8.8 (5.7 to 12.1)
45–64	493 (51.1)	334,975 (35.6)	15.5 (12.3 to 18.8)
65–84	69 (7.2)	202,419 (21.5)	−14.3 (−15.9 to −12.4)
85 and over	0 (0)	31,257 (3.3)	−3.3 (−3.4 to −2.9)
IMD (Quintiles)			
Q1 (least deprived)	143 (14.8)	238,806 (25.4)	−10.6 (−12.8 to −8.1)
Q2	137 (14.2)	194,743 (20.7)	−6.5 (−8.6 to −4.0)
Q3	177 (18.4)	190,264 (20.2)	−1.8 (−4.3 to 0.8)
Q4	230 (23.9)	170,854 (18.2)	5.7 (3.1 to 8.6)
Q5 (most deprived)	277 (28.7)	146,446 (15.6)	13.1 (10.4 to 16.2)

HIV=human immunodeficiency virus. 95%CI=95% confidence interval. IMD=index of multiple deprivation.

## Health condition groups

**Figure 1:**
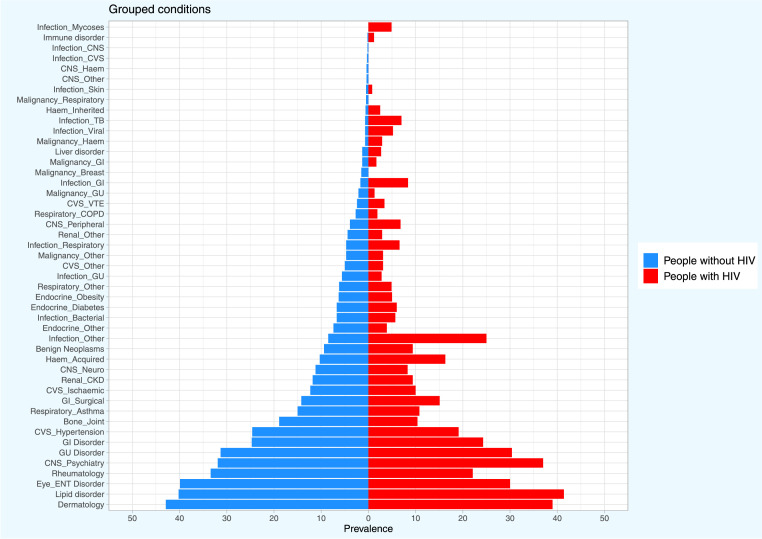
Prevalence of condition groups in adults A) with and B) without HIV. HIV=human immunodeficiency virus. TB=tuberculosis. GI=gastrointestinal. Haem=haematological. CNS=central nervous system. VTE=venous thromboembolism. GU=genitourinary/gynaecological. CKD=chronic kidney disease. CVS=cardiovascular. ENT=ear, nose and throat. COPD=chronic obstructive pulmonary disease. OR=odds ratio. 95%CI=95% confidence interval. IMD=index of multiple deprivation.

The prevalence of the 47 condition groups in adults with and without HIV is shown in Figure 1 and Table 2. Condition groups with the greatest prevalence in adults with HIV were similarly highly prevalent in those without HIV. In adults with and without HIV respectively these included: lipid (41.4% vs 40.2%), dermatological (39.0% vs 42.9%), GU (30.4% vs 31.3%), GI (24.3% vs 24.7%), and rheumatological (22.1% vs 33.4%) disorders.

**Table 2:** Prevalence and odds ratios for condition groups in adults with and without HIV.

Health condition	People with HIV, n (%) (*N* = 964)	People without HIV, n (%) (*N* = 941,113)	Unadjusted OR (95% CI)	Adjusted for age and sex OR (95% CI)	Adjusted for age, sex and IMD OR (95% CI)
Infection_Mycoses	47 (4.9)	432 (0)	111.61 (81.02–150.20)	120.86 (87.39–163.44)	114.49 (82.61–155.17)
Infection_TB	67 (7)	6147 (0.7)	11.36 (8.77–14.46)	13.55 (10.42–17.33)	12.43 (9.56–15.90)
Immune disorder	12 (1.2)	1928 (0.2)	6.14 (3.27–10.36)	7.40 (3.93–12.55)	7.50 (3.98–12.74)
Infection_Viral	50 (5.2)	6898 (0.7)	7.41 (5.50–9.75)	7.68 (5.70–10.11)	7.20 (5.34–9.48)
Infection_GI	81 (8.4)	16,096 (1.7)	5.27 (4.17–6.58)	5.74 (4.53–7.17)	5.24 (4.13–6.55)
Malignancy_Haem	28 (2.9)	6502 (0.7)	4.30 (2.88–6.14)	4.88 (3.26–7.01)	4.89 (3.26–7.02)
Haem_Inherited	24 (2.5)	6076 (0.6)	3.93 (2.55–5.76)	4.21 (2.73–6.18)	3.90 (2.52–5.72)
Infection_Other	241 (25)	80,323 (8.5)	3.57 (3.08–4.13)	4.18 (3.60–4.85)	3.81 (3.27–4.42)
Infection_CNS	<5	1710 (0.2)	2.86 (1.02–6.18)	3.07 (1.10–6.64)	2.93 (1.05–6.33)
Haem_Acquired	157 (16.3)	97,192 (10.3)	1.69 (1.42–2.00)	2.17 (1.81–2.59)	2.03 (1.69–2.42)
CNS_Peripheral	66 (6.8)	36,673 (3.9)	1.81 (1.40–2.31)	2.06 (1.59–2.64)	2.00 (1.54–2.56)
Liver disorder	26 (2.7)	12,563 (1.3)	2.05 (1.35–2.96)	2.17 (1.43–3.14)	1.96 (1.29–2.83)
CVS_VTE	33 (3.4)	22,692 (2.4)	1.43 (0.99–2.00)	1.72 (1.19–2.41)	1.62 (1.12–2.26)
Infection_Respiratory	64 (6.6)	44,052 (4.7)	1.45 (1.11–1.85)	1.71 (1.30–2.19)	1.50 (1.14–1.93)
Malignancy_GI	16 (1.7)	12,414 (1.3)	1.26 (0.74–2.00)	1.50 (0.87–2.39)	1.45 (0.84–2.31)
Infection_Skin	8 (0.8)	4628 (0.5)	1.69 (0.77–3.17)	1.56 (0.71–2.92)	1.45 (0.66–2.70)
CNS_Psychiatry	357 (37)	300,361 (31.9)	1.25 (1.10–1.43)	1.39 (1.22–1.59)	1.28 (1.12–1.46)
Benign Neoplasms	91 (9.4)	88,402 (9.4)	1.01 (0.80–1.24)	1.27 (1.01–1.58)	1.26 (1.00–1.56)
GI_Surgical	146 (15.1)	133,600 (14.2)	1.08 (0.90–1.28)	1.21 (1.01–1.44)	1.21 (1.01–1.45)
GU Disorder	293 (30.4)	294,425 (31.3)	0.96 (0.83–1.10)	1.20 (1.04–1.39)	1.18 (1.02–1.37)
GI Disorder	234 (24.3)	232,338 (24.7)	0.98 (0.84–1.13)	1.12 (0.96–1.31)	1.10 (0.94–1.28)
Lipid disorder	399 (41.4)	378,025 (40.2)	1.11 (0.93–1.32)	1.10 (0.92–1.31)	1.10 (0.92–1.31)
Malignancy_Respiratory	<5	4494 (0.5)	0.87 (0.27–2.02)	1.04 (0.32–2.43)	0.96 (0.30–2.25)
Renal_CKD	91 (9.4)	110,722 (11.8)	0.78 (0.63–0.96)	1.00 (0.78–1.25)	0.93 (0.73–1.17)
Infection_Bacterial	55 (5.7)	62,884 (6.7)	0.85 (0.64–1.10)	1.01 (0.76–1.32)	0.93 (0.70–1.21)
Dermatology	376 (39)	403,662 (42.9)	0.85 (0.75–0.97)	0.88 (0.77–1.00)	0.90 (0.79–1.03)
Endocrine_Diabetes	58 (6)	63,262 (6.7)	0.89 (0.67–1.15)	1.00 (0.75–1.30)	0.90 (0.68–1.17)
CVS_Ischaemic	96 (10)	116,163 (12.3)	0.79 (0.63–0.96)	0.92 (0.72–1.15)	0.84 (0.67–1.06)
Malignancy_Other	30 (3.1)	43,838 (4.7)	0.66 (0.45–0.93)	0.79 (0.53–1.12)	0.82 (0.55–1.17)
Endocrine_Obesity	48 (5)	59,076 (6.3)	0.78 (0.58–1.03)	0.89 (0.65–1.17)	0.80 (0.59–1.06)
Respiratory_Other	47 (4.9)	58,678 (6.2)	0.77 (0.57–1.02)	0.85 (0.62–1.13)	0.79 (0.58–1.06)
CVS_Hypertension	184 (19.1)	231,652 (24.6)	0.72 (0.61–0.85)	0.85 (0.70–1.02)	0.78 (0.65–0.94)
CNS_Neuro	80 (8.3)	105,631 (11.2)	0.72 (0.57–0.89)	0.79 (0.62–0.99)	0.78 (0.61–0.97)
Infection_CVS	<5	2889 (0.3)	0.68 (0.11–2.09)	0.79 (0.13–2.45)	0.78 (0.13–2.42)
CNS_Haem	<5	4221 (0.4)	0.69 (0.17–1.80)	0.79 (0.20–2.07)	0.75 (0.19–1.95)
Eye_ENT Disorder	289 (30)	375,502 (39.9)	0.64 (0.56–0.74)	0.70 (0.60–0.80)	0.71 (0.61–0.81)
Renal_Other	28 (2.9)	41,144 (4.4)	0.65 (0.44–0.93)	0.78 (0.52–1.12)	0.70 (0.47–1.01)
CVS_Other	30 (3.1)	46,859 (5)	0.61 (0.42–0.86)	0.71 (0.48–1.02)	0.69 (0.47–0.98)
Malignancy_GU	13 (1.3)	19,941 (2.1)	0.63 (0.35–1.04)	0.68 (0.37–1.13)	0.68 (0.37–1.14)
Respiratory_COPD	18 (1.9)	25,698 (2.7)	0.68 (0.41–1.05)	0.80 (0.48–1.25)	0.67 (0.40–1.05)
Respiratory_Asthma	104 (10.8)	141,624 (15)	0.68 (0.55–0.83)	0.66 (0.54–0.81)	0.65 (0.53–0.80)
Endocrine_Other	38 (3.9)	69,748 (7.4)	0.51 (0.36–0.70)	0.66 (0.47–0.90)	0.65 (0.46–0.89)
Rheumatology	213 (22.1)	314,717 (33.4)	0.56 (0.48–0.66)	0.62 (0.52–0.72)	0.62 (0.52–0.72)
Bone_Joint	100 (10.4)	177,465 (18.9)	0.50 (0.40–0.61)	0.58 (0.46–0.71)	0.56 (0.44–0.69)
Infection_GU	27 (2.8)	52,803 (5.6)	0.48 (0.32–0.70)	0.60 (0.39–0.86)	0.55 (0.36–0.79)
Malignancy_Breast	<5	13,999 (1.5)	0.35 (0.12–0.74)	0.53 (0.19–1.14)	0.54 (0.19–1.18)
CNS_Other	<5	3953 (0.4)	0.25 (0.01–1.09)	0.28 (0.02–1.24)	0.27 (0.02–1.21)

HIV=human immunodeficiency virus. TB=tuberculosis. GI=gastrointestinal. Neuro=neurological. Haem=haematological. CNS=central nervous system. VTE=venous thromboembolism. GU=genitourinary/gynaecological. CKD=chronic kidney disease. CVS=cardiovascular. ENT=ear, nose and throat. COPD=chronic obstructive pulmonary disease. OR=odds ratio. 95%CI=95% confidence interval. IMD=index of multiple deprivation.

**Figure 2:**
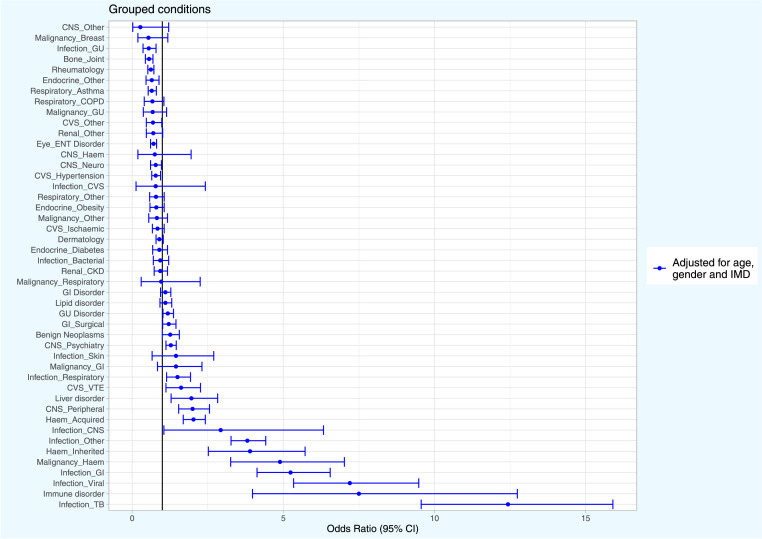
Odds ratios for 47 condition groups in adults with HIV compared to those without HIV. Infection_Mycoses 95% CI Interval: 114.49 (82.61–155.17). HIV=human immunodeficiency virus. TB=tuberculosis. GI=gastrointestinal. Haem=haematological. CNS=central nervous system. VTE=venous thromboembolism. GU=genitourinary/gynaecological. CKD=chronic kidney disease. CVS=cardiovascular. ENT=ear, nose and throat. COPD=chronic obstructive pulmonary disease. OR=odds ratio. 95%CI=95% confidence interval. IMD=index of multiple deprivation.

Following adjustment for age, sex and deprivation, 18 (37.5%) of the condition groups were significantly more likely in adults with HIV, whilst 10 (20.8%) were significantly less likely in adults with HIV (Table 2 and Figure 2). Infections were most likely to be present in adults with HIV such as mycoses (odds ratio [OR] 114.49 [95% CI 82.61–155.17]), TB infection (OR 12.43 [9.56–15.90]), viral infection (OR 7.20 [5.34–9.48]), GI infection (OR 5.24 [4.13–6.55]) and other infection type (OR 3.81 [3.27–4.42]). This was similar for haematological conditions including haematological malignancy (OR 4.89 [3.26–7.02]) and inherited haematological conditions (OR 3.90 [2.52–5.72]) By contrast, the condition groups least likely to be present in adults with HIV included: end stage renal disease (OR 0.46 [0.27–0.73]), GU infection (OR 0.55 [0.36–0.79]), bone and joint disorder (OR 0.56 [0.44–0.69]), rheumatological disorder (OR 0.62 [0.52–0.72]) and asthma (OR 0.65 [0.53–0.80]). The association between HIV status and each condition group was also evaluated with and without inclusion of deprivation in the model (Table 2 and Appendix: Figure A1). Following adjustment for deprivation, most associations were smaller than without inclusion of deprivation in the model. However, significance changed with only two condition groups following inclusion of deprivation (hypertension: OR 0.78 [0.65–0.94] without deprivation vs OR 0.85 [0.70–1.02]) with; and CVS other: OR 0.69 [0.47–0.98] vs OR 0.71 [0.48–1.02], respectively).

## Individual health conditions

### Cardiovascular conditions

The prevalence of coronary ischaemic conditions and hypertension was lower amongst adults with HIV compared to those without (Appendix: Table A4 and Appendix: Figure A2). Following adjustment the prevalence and probability of atrial fibrillation (1.2%, OR 0.37 [0.20–0.64]), stable angina (2.8%, OR 0.63 [0.41–0.91]), coronary artery disease not otherwise specified (3.4%, OR 0.52 [0.36–0.73]), and hypertension (19.1%, OR 0.78 [0.65–0.94]) were lower and less likely amongst adults with HIV compared to those without HIV, respectively. By contrast, adults with HIV had a higher prevalence and probability of pulmonary embolism (1.5%, OR 2.06 [1.15–3.36]), and raised triglycerides (9.6%, OR 1.34 [1.06–1.67]).

### Rheumatological and bone and joint conditions

The prevalence and probability of rheumatoid arthritis (<0.6%, OR 0.27 [0.05–0.84]), osteoarthritis (4.4%, OR 0.38 [0.27–0.52], wrist fracture (2.2%, OR 0.59 [0.37–0.88]), carpal tunnel syndrome (2.8%, OR 0.66 [0.44–0.96]) and enthesopathies and synovial disorders (15.1%, OR 0.65 [0.54–0.78] were all lower and less likely in adults with HIV compared to those without respectively (Appendix: Table A5 and Appendix: Figure A3). By contrast, none of the other rheumatological and bone and joint conditions were significantly more likely in adults with HIV.

### Respiratory, renal and endocrine conditions

The prevalence and probability of asthma (10.8%, OR 0.65 [0.53–0.80]), sleep apnoea (0.8%, OR 0.53 [0.24–0.99]), end-stage renal disease (1.7%, OR 0.46 [0.27–0.73]), acute kidney injury (1.6%, OR 0.45 [0.26–0.73]) and polycystic ovarian syndrome (<0.6%, OR 0.15 [0.01–0.68]) were all lower and less likely in adults with HIV compared to those without respectively (Appendix: Table A6 and Appendix: Figure A4). By contrast, none of the other respiratory, renal and endocrine conditions were significantly more likely in adults with HIV.

### GI, GU and gynaecological conditions

Amongst GI conditions, the prevalence and probability of anorectal fistula (2.5%, OR 4.22 [2.81–6.07]), anal fissure (5.4%, OR 1.99 [1.49–2.61]), gastritis and duodenitis (6.3%, OR 1.32 [1.00–1.69]), and oesophagitis and oesophageal ulcer (8.4%, OR 1.28 [1.00–1.60]) was higher and more likely in adults with HIV whilst the prevalence and probability of diverticular disease (0.9%, OR 0.31 [0.15–0.57]) and cholecystitis (0.7%, OR 0.36 [0.15–0.70]) was lower and less likely (Appendix: Table A7 and Appendix: Figure A5). Amongst GU and gynaecological conditions, the prevalence and probability of male infertility (3.0%, OR 2.23 [1.50–3.16]), female infertility (2.9%, OR 2.01 [1.34–2.88]), and erectile dysfunction (11.2%, OR 1.98 [1.59–2.46]) was higher and more likely in adults with HIV, while the prevalence and probability of female genital prolapse (0.7%, OR 0.37 [0.16–0.73]) was lower and less likely.

### Special senses

The prevalence and probability of hearing loss (4.9%, OR 0.51 [0.38–0.68]), glaucoma (1.1%, OR 0.40 [0.20–0.69]), macular degeneration (<0.6%, OR 0.30 [0.09–0.70]) and cataract (3.5%, OR 0.50 [0.34–0.70]) were all lower and less likely in adults with HIV compared to those without (Appendix: Table A8 and Appendix: Figure A6). By contrast, the prevalence and probability of posterior uveitis (1.0%, OR 16.83 [8.36–29.88]), eye infections (0.6%, OR 8.61 [3.40–17.61]) and seberrhoic dermatitis (7.0%, OR 1.37 [1.06–1.74]) were all higher and more likely in adults with HIV respectively.

### CNS-related conditions

The prevalence and probability of Bell's palsy (2.8%, OR 3.08 [2.04–4.42]), peripheral neuropathy (3.7%, OR 1.79 [1.25–2.46]), bipolar affective disorder and mania (1.1%, OR 2.93 [1.52–5.05]), substance misuse (5.2%, OR 2.45 [1.81–3.23]), alcohol problems (7.4%, OR 1.64 [1.27–2.07]) and depression (27.1%, OR 1.49 [1.28–1.71]) were higher and more likely in adults with HIV respectively (Appendix: Table A9 and Appendix: Figure A7). By contrast, none of the conditions were significantly less likely in adults with HIV apart from migraine (6.2%, OR 0.76 [0.58–0.98]).

### Benign neoplasms and malignancies

The prevalence and probability of cervical intra-epithelial neoplasia (3.5%, OR 4.75 [3.28–6.66]), cervical malignancy (<0.6%, OR 4.64 [1.15–12.15]), Hodgkin's lymphoma (<0.6%, OR 4.66 [1.15–12.16]) and non-Hodgkin's lymphoma (2.3%, OR 11.92 [7.53–17.86]) were all higher and more likely in adults with HIV compared to those without respectively (Appendix: Table A10 and Appendix: Figure A8). By contrast, none of the other benign neoplasms and malignancies were significantly less likely in adults with HIV.

### Haematological conditions

The prevalence and probability of aplastic anaemia (0.6%, OR 16.70 [6.58–34.34]), haemolytic anaemia (<0.6%, OR 4.82 [1.49–11.28]), agranulocytosis (2.8%, OR 4.31 [2.86–6.20]), primary (0.7%, OR 4.26 [1.82–8.28]) and secondary (2.4%, OR 4.30 [2.75–6.36]) thrombocytopenia, folate deficiency (1.2%, OR 2.65 [1.41–4.49]) and sickle cell anaemia (<0.6%, OR 6.49 [1.60–17.00]) was higher and more likely in adults with HIV compared to those without respectively (Appendix: Table A11 and Appendix: Figure A9). By contrast, none of the other haematological conditions was significantly less likely in adults with HIV.

### Infections

The prevalence and probability of many infection types was greater in adults with HIV compared to those without (Appendix: Table A12 and Appendix: Figure A10). Individual infections with the strongest association (not otherwise aforementioned in the condition group section) included infection of the anal and rectal regions (1.9%, OR 32.39 [19.39–50.61]), chronic viral hepatitis (4.9%, OR 22.17 [16.23–29.53]), meningitis (<0.6%, OR 7.10 [1.17–22.20]), parasitic infections (1.1%, OR 14.98 [7.72–25.96]), and infection of the bone and joints (<0.6%, OR 4.61 [1.14–12.05]). By contrast, only urinary tract infection was significantly less likely in adults with HIV (2.1%, OR 0.43 [0.27–0.66]).

### Liver disorders

The prevalence and probability of liver fibrosis, sclerosis and cirrhosis was greater in adults with HIV compared to those without (1.6%, OR 3.23 [1.85–5.20], Appendix: Table A13).

## Discussion

To our knowledge, this is the first large-scale analysis of comorbid health conditions in adults with HIV compared to the general population in England and we observed several potentially important findings. We demonstrated that highly prevalent conditions in adults without HIV are still highly prevalent in those with HIV, such as hypertension, lipid disorders, GI disorders and rheumatological disorders. We also observed that adults with HIV still had a high probability of infection including mycoses, TB, parasitic and viral infections. Despite advances in HIV treatment and care that are associated with a lower mortality in adults with HIV, our findings reflect the expected immune dysfunction in people with HIV.

Previous studies, typically of long term cohorts, have found increased risk of cardiovascular disease amongst people with HIV.^19^^,^^20^ In our cross-sectional study focusing on 2015, overall probability of ischaemic cardiovascular disease was not elevated, reflecting perhaps the accumulation of awareness about risks, their pro-active management, earlier initiation of HIV therapy, and availability of HIV therapies with fewer metabolic side effects than in the past. We also observed that adults with HIV had a lower probability of rheumatoid arthritis. A reduced incidence of rheumatoid arthritis in people with HIV has recently been described in a US study.^21^ People with HIV and incident rheumatoid arthritis also had less positive autoantibody profiles and were prescribed disease modifying anti-rheumatic drugs less frequently than controls. This is interesting because an increased frequency of autoantibodies and impaired immune tolerance are shared features between rheumatoid arthritis and HIV.^22^^,^^23^ Adults with HIV also had a lower probability of asthma where data are more limited and conflicting. For instance, a 2011 cohort study suggested that HIV status did not influence asthma rates whilst a cross-sectional from 2014 reported that people with HIV had increased asthma prevalence.^9^^,^^24^ Studies measuring differences in bronchial hyper-reactivity in people with asthma according to HIV status have also been conflicting.^25^^,^^26^ Furthermore, whilst asthma prevalence was 20.6% amongst people with HIV in a US study, it involved only a small selected population without a control group.^27^

It is recognised that people with HIV are at increased risk of developing several neurological, haematological and malignant conditions including Bell's palsy, peripheral neuropathy, lymphoma and cervical malignancy. We similarly observed these associations in our study. We also observed a greater risk of pulmonary embolism amongst adults with HIV. This has previously been reported and could be related to the procoagulant state and increased risk of venous thromboembolism rates associated with other infections.^28^^,^^29^ However, we also observed other associations including male and female infertility that may be related to hypogonadism for example.^30^^,^^31^

Our study has several strengths. We make comparisons between adults with HIV and the entire general population within the database, likely to provide more accurate measures of prevalence than using a sample of controls. We were also able to adjust for confounding by deprivation, which often resulted in smaller associations suggesting socioeconomic gradient may be an important factor for some health conditions with the largest difference evident for infection groups.^32^ Our methodological approach is similar to that used to study the prevalence of comorbidity in other disease areas and we observe similar associations expected with many conditions supporting the validity of results.^33^, ^34^, ^35^ Our study also has some limitations. Our study is cross-sectional in nature and does not provide information on the temporal relationship between HIV and each health condition. Some health conditions will still be active at the index date (certain comorbidities such as IHD and diabetes, for example) whilst more acute health conditions will have occurred before the index date and will have resolved (infections and pulmonary embolism, for example). Similarly, some associations may be related to risk factors for contracting HIV whilst others may be as a result chronic HIV infection or treatment. Despite the large database, the number of HIV patients in our cross-section was limited which may reflect less primary care engagement by some sections of adults with HIV or geographical spread of CPRD-contributing practices. This meant examining associations with rarer conditions was not possible. This study is dependant upon the quality of data recording. It is possible that some adults with HIV may not have had a record of HIV in their primary care records although we also included patients with HIV codes from HES. Furthermore, CPRD does not capture secondary care blood tests where some HIV cases will be diagnosed. It is uncertain whether such patients would have a different prevalence of health conditions. However, the estimated UK prevalence of known HIV in 2015 was 0.14% compared to 0.1% in our population that is not too dissimilar. Whilst this may result in some adults with HIV being misclassified into the general population such numbers would be small compared to the general population and therefore unlikely to impact our findings. This may also be similar for the recording of other health conditions. However, CPRD data is regarded as being generalisable, of high quality and validity, and has been used extensively for research. We adjusted for confounding factors of age, sex and deprivation. Despite this, we noted there were no patients with HIV aged 85 years and over potentially influencing the generalisability of our results to older patients with HIV that remains an area for future evaluation. However, this largely reflects the current epidemiology of HIV.^2^

Morbidity and mortality from HIV have significantly improved due to improved care pathways and earlier use of anti-retroviral therapy meaning HIV patients are more likely to develop other health conditions. This has important implications for the management of these other health conditions in such patients. Evidence in many guidelines for single health conditions is often drawn from people without multimorbidity and who take fewer prescribed medicines. Individual treatment recommendations from such guidelines therefore require further consideration around the benefits and risks in people with multimorbidity to avoid unnecessary harm for example with drug-drug interaction. An approach that takes into account multimorbidity and/or risk factors for developing other health conditions within guidelines is therefore required and our analysis may help to inform such planning.^5^ Another area of future work would be developing and evaluating different approaches to health care provision and service delivery in HIV care that optimizes the management of other health conditions.

Concomitant morbidity is common in adults with HIV. Using an established list of conditions frequently encountered in UK health services, we found that many were more likely to afflict adults with HIV than those without HIV. Accordingly, in order to assure holistic care, specialist and primary care providers need close integration in the particular context of the UK, where HIV care is exclusively delivered by secondary care.

## Declaration of interests

We declare no competing interests.

## Appendix Supplementary materials

Image, application 1
