# Supplementary material for: Health conditions in adults with HIV compared with the general population: A population-based cross-sectional analysis
Source: eClinicalMedicine. 2022 Apr 21;47:101392. doi: 10.1016/j.eclinm.2022.101392 (PMC9046106; doi:10.1016/j.eclinm.2022.101392)
Supplement: Supplementary file 1 [file mmc1.pdf]

HEALTH CONDITIONS IN ADULTS WITH HIV COMPARED WITH THE GENERAL POPULATION: POPULATION-BASED CROSS-SECTIONAL ANALYSIS

APPENDIX

Daniel R. Morales, [1]Division of Population Health and Genomics, University of Dundee, UK.
[2]Department of Public Health, University of Southern Denmark, Denmark

David Moreno Martos, Division of Population Health and Genomics, University of Dundee, UK

Nashaba Matin, Barts Health NHS Trust, Grahame Hayton Unit, Royal London Hospital, London, UK

Patricia McGettigan, William Harvey Research Institute, Queen Mary University of London, UK

Contents

Table A1. Primary care codes to identify patients with HIV. 4

Table A2. ICD-10 codes to identify patients with HIV from hospitalisations (HES). 7

Table A3. Proportion of patients with a previous lipid and estimated glomerular filtration rate blood test. 8

Figure A1. Odds ratios for 47 health condition groups in adults with HIV compared to those without HIV before (blue) and after (red) adjustment for deprivation. 9

Table A4. Prevalence and odds ratios of individual cardiovascular conditions in adults with HIV compared to those without HIV. 10

Figure A2. Prevalence (A) and odds ratios (B) of individual cardiovascular conditions in adults with HIV compared to those without HIV. 11

Table A5. Prevalence and odds ratios of individual rheumatological and other bone and joint conditions in adults with HIV compared to those without HIV. 12

Figure A3. Prevalence (A) and odds ratios (B) of individual rheumatological and other bone and joint conditions in adults with HIV compared to those without HIV. 13

Table A6. Prevalence and odds ratios of individual respiratory, renal and endocrine conditions in adults with HIV compared to those without HIV. 14

Figure A4. Prevalence (A) and odds ratios (B) of individual respiratory, renal and endocrine conditions in adults with HIV compared to those without HIV. 15

Table A7. Prevalence and odds ratios of individual GI, gynaecological and GU conditions in adults with HIV compared to those without HIV. 16

Figure A5. Prevalence and odds ratios of individual GI, gynaecological and GU conditions in adults with HIV compared to those without HIV. 17

Table A8. Prevalence and odds ratios of individual conditions related to the special senses in adults with HIV compared to those without HIV. 18

Figure A6. Prevalence and odds ratios of individual conditions related to the special senses in adults with HIV compared to those without HIV. 19

Table A9. Prevalence and odds ratios of individual CNS-related conditions in adults with HIV compared to those without HIV. 20

Figure A7. Prevalence and odds ratios of individual CNS-related conditions in adults with HIV compared to those without HIV. 21

Table A10. Prevalence and odds ratios of individual benign neoplasms and malignant conditions in adults with HIV compared to those without HIV. 22

Figure A8. Prevalence and odds ratios of individual benign neoplasms and malignant conditions in adults with HIV compared to those without HIV. 23

Table A11. Prevalence and odds ratios of individual haematological conditions in adults with HIV compared to those without HIV. 24

Figure A9. Prevalence and odds ratios of individual haematological conditions in adults with HIV compared to those without HIV. 25

Table A12. Prevalence and odds ratios of individual infections in adults with HIV compared to those without HIV. 26
Figure A10. Prevalence and odds ratios of individual infections in adults with HIV compared to those without HIV. 27
Table A13. Prevalence and odds ratios of individual liver disorders in adults with HIV compared to those without HIV. 28

Table A1. Primary care codes to identify patients with HIV.

Medcode	Read code	Description
2835	43C3.11	HIV positive
23763	65QA.00	AIDS carrier
98966	66j..00	Human immunodeficiency virus monitoring
101191	66j0.00	Human immunodeficiency virus annual review
105040	9kl..00	HIV pos gen health check serv declind - enhanc service admin
111971	9kl..11	HIV positive general health check service declined
108385	9mN..00	Human immunodeficiency virus infection monitoring invitation
109327	9mN0.00	HIV infection monitoring telephone invitation
111972	9mN0000	HIV infection monitoring first telephone invitation
111973	9mN0100	HIV infection monitoring second telephone invitation
111974	9mN0200	HIV infection monitoring third telephone invitation
23770	A788.00	Acquired immune deficiency syndrome
9130	A788.11	Human immunodeficiency virus infection
58857	A788000	Acute human immunodeficiency virus infection
58859	A788100	Asymptomatic human immunodeficiency virus infection
69766	A788200	HIV infection with persistent generalised lymphadenopathy
70869	A788300	Human immunodeficiency virus with constitutional disease
53636	A788400	Human immunodeficiency virus with neurological disease
70528	A788500	Human immunodeficiency virus with secondary infection
101836	A788600	Human immunodeficiency virus with secondary cancers
101836	A788600	Human immunodeficiency virus with secondary cancers
47632	A788U00	HIV disease result/haematological+immunologic abnorms
111979	A788V00	HIV disease resulting in multiple diseases CE
111979	A788V00	HIV disease resulting in multiple diseases CE
67575	A788W00	HIV disease resulting in unspecified malignant neoplasm
71450	A788X00	HIV disease resulting/unspcf infectious+parasitic disease
62891	A788y00	Human immunodeficiency virus with other clinical findings
44303	A789.00	Human immunodef virus resulting in other disease
37006	A789000	HIV disease resulting in mycobacterial infection
66368	A789100	HIV disease resulting in cytomegaloviral disease
23951	A789200	HIV disease resulting in candidiasis

27641	A789300	HIV disease resulting in Pneumocystis carinii pneumonia
104717	A789311	HIV disease resulting in Pneumocystis jirovecii pneumonia
104717	A789311	HIV disease resulting in Pneumocystis jirovecii pneumonia
50076	A789400	HIV disease resulting in multiple infections
27853	A789500	HIV disease resulting in Kaposi's sarcoma
108054	A789511	HIV disease resulting in Kaposi sarcoma
108054	A789511	HIV disease resulting in Kaposi sarcoma
44617	A789600	HIV disease resulting in Burkitt's lymphoma
111980	A789611	HIV disease resulting in Burkitt lymphoma
111980	A789611	HIV disease resulting in Burkitt lymphoma
66367	A789700	HIV dis resulting oth types of non-Hodgkin's lymphoma
111981	A789711	HIV disease resulting in other types of non-Hodgkin lymphoma
111981	A789711	HIV disease resulting in other types of non-Hodgkin lymphoma
105324	A789800	HIV disease resulting in multiple malignant neoplasms
105324	A789800	HIV disease resulting in multiple malignant neoplasms
65117	A789900	HIV disease resulting in lymphoid interstitial pneumonitis
8281	A789A00	HIV disease resulting in wasting syndrome
51708	A789X00	HIV dis reslt/oth mal neopl/lymph
112030	AyuC000	[X]HIV disease resulting in other bacterial infections
112030	AyuC000	[X]HIV disease resulting in other bacterial infections
107807	AyuC100	[X]HIV disease resulting in other viral infections
107807	AyuC100	[X]HIV disease resulting in other viral infections
112031	AyuC200	[X]HIV disease resulting in other mycoses
112031	AyuC200	[X]HIV disease resulting in other mycoses
102117	AyuC300	[X]HIV disease resulting in multiple infections
102117	AyuC300	[X]HIV disease resulting in multiple infections
104134	AyuC400	[X]HIV disease resulting/other infectious+parasitic diseases
104134	AyuC400	[X]HIV disease resulting/other infectious+parasitic diseases
112032	AyuC500	[X]HIV disease resulting/unspcf infectious+parasitic disease
112032	AyuC500	[X]HIV disease resulting/unspcf infectious+parasitic disease
69767	AyuC600	[X]HIV disease resulting in other non-Hodgkin's lymphoma
112033	AyuC611	[X]HIV disease resulting in other non-Hodgkin lymphoma
112033	AyuC611	[X]HIV disease resulting in other non-Hodgkin lymphoma

112034	AyuC700	[X]HIV dis reslt/oth mal neopl/lymph
112034	AyuC700	[X]HIV dis reslt/oth mal neopl/lymph
112035	AyuC800	[X]HIV disease resulting in other malignant neoplasms
112035	AyuC800	[X]HIV disease resulting in other malignant neoplasms
112036	AyuC900	[X]HIV disease resulting in unspecified malignant neoplasm
112036	AyuC900	[X]HIV disease resulting in unspecified malignant neoplasm
112037	AyuCA00	[X]HIV disease resulting in multiple diseases CE
112037	AyuCA00	[X]HIV disease resulting in multiple diseases CE
96751	AyuCB00	[X]HIV disease result/haematological+immunologic abnorms
102252	AyuCC00	[X]HIV disease resulting in other specified conditions
102252	AyuCC00	[X]HIV disease resulting in other specified conditions
100769	AyuCD00	[X]Unspecified human immunodeficiency virus [HIV] disease
100769	AyuCD00	[X]Unspecified human immunodeficiency virus [HIV] disease
41185	Eu02400	[X]Dementia in human immunodef virus [HIV] disease
104466	L179.00	HIV disease complicating pregnancy childbirth puerperium
44288	R109.00	[D]Laboratory evidence of human immunodeficiency virus [HIV]
24872	ZV01A00	[V]Asymptomatic human immunodeficency virus infection status

Table A2. ICD-10 codes to identify patients with HIV from hospitalisations (HES).

ICD-10 code	ICD-10 term
B200	HIV disease resulting in mycobacterial infection
B201	HIV disease resulting in other bacterial infections
B202	HIV disease resulting in cytomegaloviral disease
B203	HIV disease resulting in other viral infections
B204	HIV disease resulting in candidiasis
B205	HIV disease resulting in other mycoses
B207	HIV disease resulting in multiple infections
B208	HIV disease resulting in other infectious and parasitic diseases
B209	HIV disease resulting in unspecified infectious or parasitic disease
B212	HIV disease resulting in other types of non-Hodgkin lymphoma
B213	HIV disease resulting in other malignant neoplasms of lymphoid, haematopoietic and related tissue
B217	HIV disease resulting in multiple malignant neoplasms
B218	HIV disease resulting in other malignant neoplasms
B219	HIV disease resulting in unspecified malignant neoplasm
B221	HIV disease resulting in lymphoid interstitial pneumonitis
B227	HIV disease resulting in multiple diseases classified elsewhere
B23	Human immunodeficiency virus [HIV] disease resulting in other conditions
B24	Unspecified human immunodeficiency virus [HIV] disease
F024	Dementia in human immunodeficiency virus [HIV] disease
R75	Laboratory evidence of human immunodeficiency virus [HIV]
Z21	Asymptomatic human immunodeficiency virus [HIV] infection status
B206	HIV disease resulting in Pneumocystis jirovecii pneumonia
B210	HIV disease resulting in Kaposi sarcoma
B211	HIV disease resulting in Burkitt lymphoma
B220	HIV disease resulting in encephalopathy
B222	HIV disease resulting in wasting syndrome

Table A3. Proportion of patients with a previous lipid and estimated glomerular filtration rate blood test.

	**People with HIV**	**People without HIV**
Total cholesterol	391 (40.6%)	374563 (39.8%)
LDL-C	441 (45.7%)	404679 (43%)
HDL-C	489 (50.7%)	451734 (48%)
Triglycerides	476 (49.4%)	444205 (47.2%)
eGFR*	327 (33.9%)	307396 (32.7%)

Chronic kidney disease defined using codes and eGFR. Lipids defined biochemically only.
LDL-C=Low density lipoprotein cholesterol. HDL-C=High density lipoprotein cholesterol.
eGFR=estimated glomerular filtration rate.

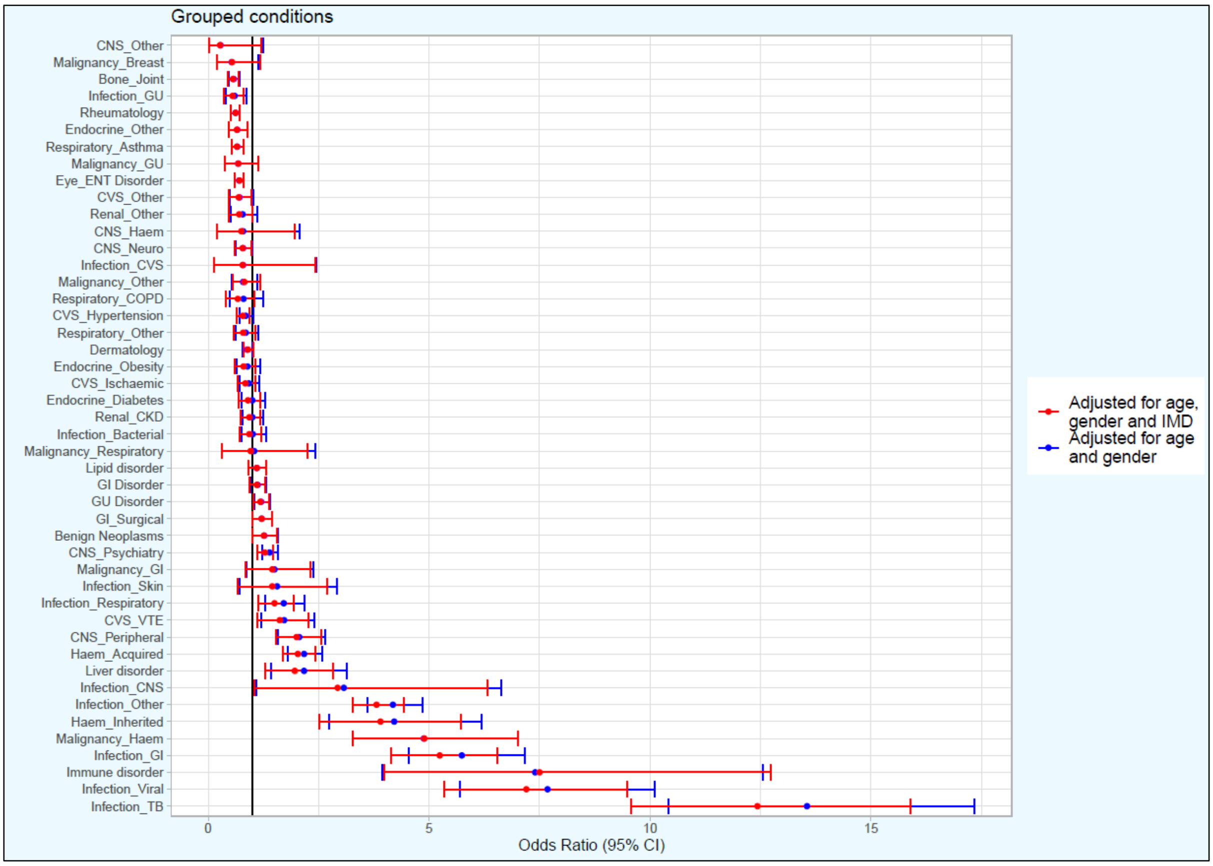

Figure A1. Odds ratios for 47 health condition groups in adults with HIV compared to those without HIV before (blue) and after (red) adjustment for deprivation.

HIV=human immunodeficiency virus. TB=tuberculosis. GI=gastrointestinal. Haem=haematological. CNS=central nervous system. VTE=venous thromboembolism. GU=genitourinary/gynaecological. CKD=chronic kidney disease. CVS=cardiovascular. ENT=ear, nose and throat. COPD=chronic obstructive pulmonary disease. OR=odds ratio. 95%CI=95% confidence interval. IMD=index of multiple deprivation.

Table A4. Prevalence and odds ratios of individual cardiovascular conditions in adults with HIV compared to those without HIV.

Health condition	People with HIV, n (%) (N=964)	People without HIV,n (%) (N=941113)	Unadjusted OR (95% CI)	Adjusted for age and sex OR (95% CI)	Adjusted for age, sex and IMD OR (95% CI)
**CVS Ischaemic**					
Atrial fibrillation	12 (1.2)	34326 (3.6)	0.33 (0.18-0.56)	0.39 (0.21-0.66)	0.37 (0.20-0.64)
Coronary heart disease not otherwise specified	33 (3.4)	60336 (6.4)	0.52 (0.36-0.72)	0.57 (0.39-0.80)	0.52 (0.36-0.73)
Heart failure	14 (1.5)	11921 (1.3)	1.15 (0.64-1.87)	1.39 (0.78-2.29)	1.25 (0.70-2.06)
Ischaemic stroke	8 (0.8)	14810 (1.6)	0.52 (0.24-0.98)	0.63 (0.28-1.18)	0.58 (0.26-1.09)
Myocardial infarction	24 (2.5)	28733 (3.1)	0.81 (0.53-1.19)	0.90 (0.58-1.34)	0.81 (0.52-1.20)
Peripheral arterial disease	17 (1.8)	12552 (1.3)	1.33 (0.79-2.07)	1.53 (0.90-2.40)	1.33 (0.79-2.10)
Stable angina	27 (2.8)	42367 (4.5)	0.61 (0.41-0.88)	0.70 (0.46-1.01)	0.63 (0.41-0.91)
Stroke NOS	21 (2.2)	18221 (1.9)	1.13 (0.71-1.69)	1.39 (0.87-2.11)	1.27 (0.79-1.93)
Transient ischaemic attack	12 (1.2)	16636 (1.8)	0.70 (0.37-1.18)	0.87 (0.46-1.47)	0.81 (0.43-1.38)
Unstable Angina	10 (1)	12363 (1.3)	0.79 (0.39-1.39)	0.90 (0.45-1.59)	0.80 (0.40-1.43)
**CVS VTE**					
Pulmonary embolism	14 (1.5)	7717 (0.8)	1.78 (1.00-2.90)	2.16 (1.21-3.53)	2.06 (1.15-3.36)
Venous thromboembolic disease (Excl PE)	21 (2.2)	17037 (1.8)	1.21 (0.76-1.81)	1.43 (0.89-2.15)	1.33 (0.84-2.01)
**CVS Hypertension**					
Hypertension	184 (19.1)	231652 (24.6)	0.72 (0.61-0.85)	0.85 (0.70-1.02)	0.78 (0.65-0.94)
**Lipid Disorder**					
Low HDL-C	131 (13.6)	77260 (8.2)	1.89 (1.55-2.29)	1.60 (1.30-1.95)	1.49 (1.22-1.82)
Raised cholesterol	268 (27.8)	290470 (30.9)	0.84 (0.71-0.98)	0.87 (0.74-1.03)	0.91 (0.77-1.07)
Raised LDL-C	217 (22.5)	249594 (26.5)	0.81 (0.68-0.97)	0.81 (0.67-0.96)	0.83 (0.69-0.99)
Raised Triglycerides	93 (9.6)	65328 (6.9)	1.56 (1.24-1.94)	1.43 (1.13-1.78)	1.34 (1.06-1.67)
**CVS Other**					
Abdominal aortic aneurysm	<5	1678 (0.2)	0.58 (0.03-2.56)	0.64 (0.04-2.85)	0.60 (0.03-2.66)
Atrioventricular block, complete	<5	2578 (0.3)	1.14 (0.28-2.96)	1.36 (0.34-3.56)	1.30 (0.32-3.40)
Atrioventricular block, first degree	<5	5308 (0.6)	0.18 (0.01-0.81)	0.22 (0.01-0.96)	0.21 (0.01-0.91)
Atrioventricular block, second degree	<5	1738 (0.2)	0.56 (0.03-2.48)	0.66 (0.04-2.90)	0.63 (0.04-2.80)
CVD (SA+UA+MI+HF+Other)	51 (6.1)	75523 (8)	0.75 (0.57-0.96)	0.85 (0.64-1.12)	0.78 (0.59-1.02)
Dilated cardiomyopathy	<5	1867 (0.2)	1.57 (0.39-4.09)	1.62 (0.40-4.23)	1.56 (0.39-4.06)
Left bundle branch block	<5	7388 (0.8)	0.26 (0.04-0.81)	0.32 (0.05-1.00)	0.30 (0.05-0.93)
Nonrheumatic aortic valve disorders	6 (0.6)	5882 (0.6)	1.00 (0.39-2.03)	1.22 (0.48-2.49)	1.21 (0.48-2.48)
Nonrheumatic mitral valve disorders	<5	5452 (0.6)	0.54 (0.13-1.39)	0.66 (0.16-1.71)	0.70 (0.17-1.81)
Other Cardiomyopathy	<5	2652 (0.3)	1.84 (0.66-3.98)	1.98 (0.71-4.27)	1.89 (0.68-4.09)
Pericardial effusion (noninflammatory)	<5	2481 (0.3)	1.97 (0.71-4.26)	2.28 (0.81-4.94)	2.17 (0.77-4.68)
Right bundle branch block	<5	8314 (0.9)	0.23 (0.04-0.72)	0.26 (0.04-0.81)	0.25 (0.04-0.77)
Supraventricular tachycardia	<5	8656 (0.9)	0.34 (0.08-0.87)	0.39 (0.10-1.01)	0.39 (0.10-1.01)
Ventricular tachycardia	<5	2497 (0.3)	0.78 (0.13-2.42)	0.85 (0.14-2.63)	0.81 (0.13-2.51)

HIV=human immunodeficiency virus. 95%CI=95% confidence interval. OR=odds ratio. IMD = index of multiple deprivation. CVD = Cardiovascular disease (composite of stable angina, unstable angina, myocardial infarction, heart failure or coronary heart disease not otherwise specified). VTE=venous thromboembolic. PE = pulmonary embolism. HDL = high density lipoprotein. LDL = low density lipoprotein.

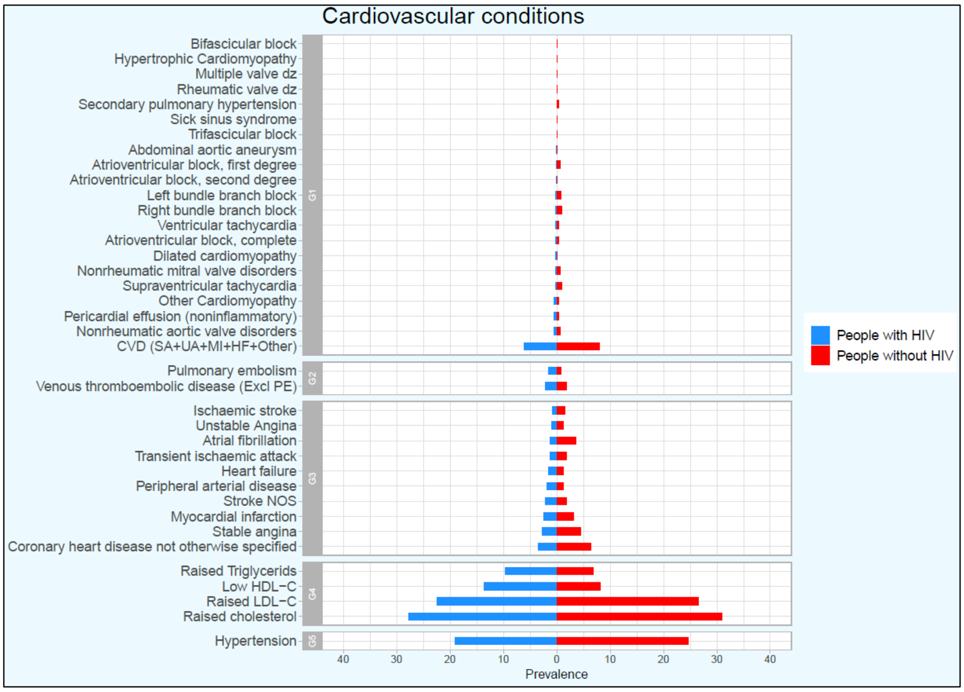

A.

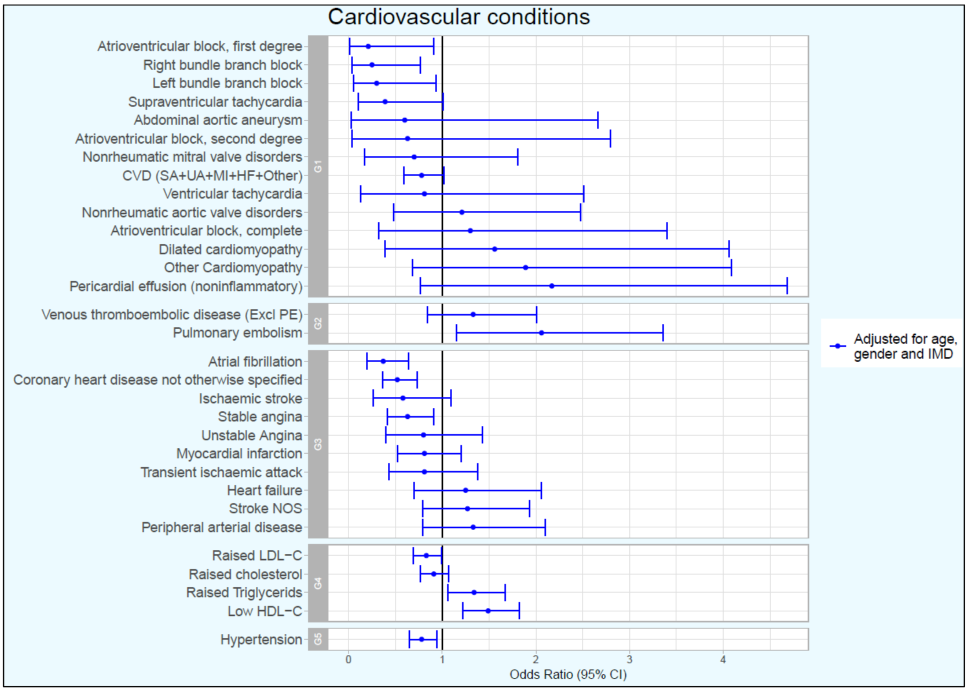

B.

Figure A2. Prevalence (A) and odds ratios (B) of individual cardiovascular conditions in adults with HIV compared to those without HIV.

CVD= cardiovascular disease. SA = stable angina. UA = unstable angina. MI = myocardial infarction. HF = heart failure. Other = Coronary heart disease not otherwise specified. PE = pulmonary embolism. LDL = low density lipoprotein. HDL = high density lipoprotein. IMD = index of multiple deprivation.

G1 = CVS_Other. G2 =CVS_VTE, G3 = CVS_Ischaemic, G4 = Lipid disorder, G5 = CVS_Hypertension.

Table A5. Prevalence and odds ratios of individual rheumatological and other bone and joint conditions in adults with HIV compared to those without HIV.

Health condition	People with HIV, n (%) (N=964)	People without HIV, n (%) (N=941113)	Unadjusted OR (95% CI)	Adjusted for age and sex OR (95% CI)	Adjusted for age, sex and IMD OR (95% CI)
**Rheumatological**					
Ankylosing spondylitis	<5	2464 (0.3)	0.40 (0.02-1.74)	0.39 (0.02-1.74)	0.39 (0.02-1.72)
Carpal tunnel syndrome	27 (2.8)	45979 (4.9)	0.56 (0.37-0.80)	0.68 (0.45-0.99)	0.66 (0.44-0.96)
Enthesopathies & synovial disorders	146 (15.1)	219958 (23.4)	0.59 (0.49-0.70)	0.64 (0.53-0.76)	0.65 (0.54-0.78)
Giant Cell arteritis	<5	1969 (0.2)	0.50 (0.03-2.18)	0.69 (0.04-3.04)	0.66 (0.04-2.92)
Gout	25 (2.6)	32864 (3.5)	0.74 (0.48-1.07)	0.73 (0.48-1.07)	0.73 (0.48-1.07)
Juvenile arthritis	<5	545 (0.1)	1.79 (0.10-7.92)	1.71 (0.10-7.55)	1.73 (0.10-7.64)
Lupus erythematosus (local and systemic)	<5	1517 (0.2)	1.29 (0.21-3.99)	1.64 (0.27-5.10)	1.58 (0.26-4.90)
Polymyalgia Rheumatica	<5	9371 (1)	0.31 (0.08-0.81)	0.43 (0.11-1.12)	0.43 (0.11-1.13)
Postinfective and reactive arthropathies	<5	1138 (0.1)	2.58 (0.64-6.71)	2.60 (0.64-6.77)	2.87 (0.71-7.49)
Postviral fatigue syndrome, neurasthenia and fibromyalgia	11 (1.1)	18671 (2)	0.57 (0.30-0.98)	0.68 (0.35-1.17)	0.63 (0.33-1.09)
Psoriatic arthropathy	<5	3196 (0.3)	0.30 (0.02-1.34)	0.33 (0.02-1.44)	0.33 (0.02-1.45)
Raynaud's syndrome	10 (1)	12565 (1.3)	0.77 (0.39-1.36)	0.90 (0.45-1.59)	0.91 (0.45-1.60)
Rheumatoid Arthritis	<5	8569 (0.9)	0.23 (0.04-0.70)	0.28 (0.05-0.88)	0.27 (0.05-0.84)
Scoliosis	<5	6390 (0.7)	0.46 (0.11-1.19)	0.50 (0.12-1.30)	0.52 (0.13-1.35)
Spondylolisthesis	<5	5256 (0.6)	0.37 (0.06-1.15)	0.45 (0.07-1.40)	0.45 (0.07-1.40)
Spondylosis	13 (1.3)	54088 (5.7)	0.22 (0.12-0.37)	0.27 (0.15-0.45)	0.25 (0.14-0.41)
**Bone and Joint**					
Collapsed vertebra	<5	3878 (0.4)	0.75 (0.19-1.96)	0.98 (0.24-2.55)	0.91 (0.23-2.38)
Fracture of hip	8 (0.8)	14045 (1.5)	0.55 (0.25-1.03)	0.76 (0.35-1.44)	0.71 (0.32-1.34)
Fracture of wrist	21 (2.2)	35563 (3.8)	0.57 (0.36-0.85)	0.59 (0.37-0.89)	0.59 (0.37-0.88)
Intervertebral disc disorders	21 (2.2)	26089 (2.8)	0.78 (0.49-1.17)	0.85 (0.53-1.27)	0.87 (0.55-1.31)
Osteoarthritis (excl spine)	42 (4.4)	110892 (11.8)	0.34 (0.25-0.46)	0.40 (0.29-0.55)	0.38 (0.27-0.52)
Osteoporosis	25 (2.6)	26754 (2.8)	0.91 (0.60-1.32)	1.41 (0.91-2.08)	1.38 (0.89-2.03)

HIV=human immunodeficiency virus. 95%CI=95% confidence interval. OR=odds ratio. IMD = index of multiple deprivation.

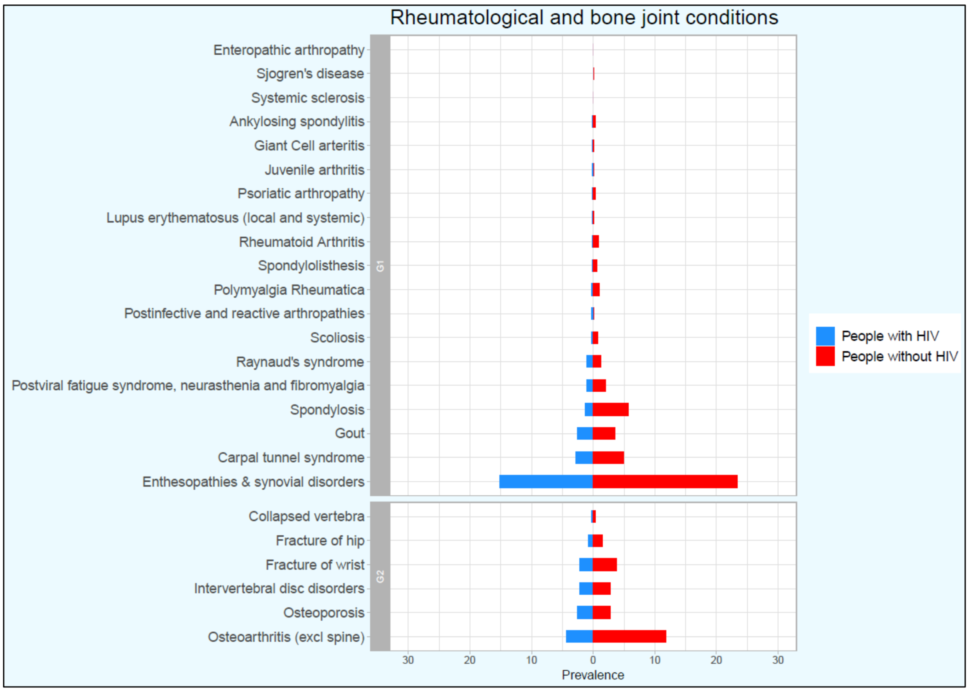

A.

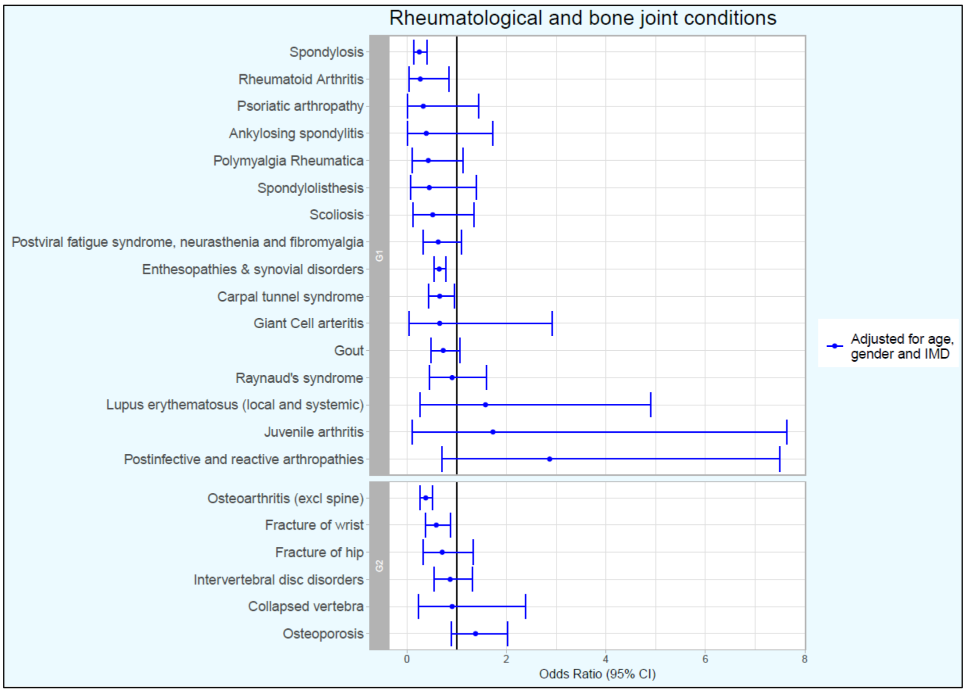

B.

Figure A3. Prevalence (A) and odds ratios (B) of individual rheumatological and other bone and joint conditions in adults with HIV compared to those without HIV.

HIV=human immunodeficiency virus. 95%CI=95% confidence interval. OR=odds ratio. IMD = index of multiple deprivation.

G1 = Rheumatology, G2 = Bone_Joint.

Table A6. Prevalence and odds ratios of individual respiratory, renal and endocrine conditions in adults with HIV compared to those without HIV.

Health condition	People with HIV, n (%) (N=964)	People without HIV, n (%) (N=941113)	Unadjusted OR (95% CI)	Adjusted for age and sex OR (95% CI)	Adjusted for age, sex and IMD OR (95% CI)
**Respiratory Asthma**					
Asthma	104 (10.8)	141624 (15)	0.68 (0.55-0.83)	0.66 (0.54-0.81)	0.65 (0.53-0.80)
**Respiratory COPD**					
COPD	18 (1.9)	25698 (2.7)	0.68 (0.41-1.05)	0.80 (0.48-1.25)	0.67 (0.40-1.05)
**Respiratory Other**					
Aspiration pneumonitis	<5	5298 (0.6)	0.37 (0.06-1.14)	0.43 (0.07-1.34)	0.39 (0.07-1.22)
Bronchiectasis	<5	6984 (0.7)	0.70 (0.25-1.50)	0.85 (0.30-1.84)	0.80 (0.29-1.74)
Other interstitial pulmonary diseases with fibrosis	<5	3346 (0.4)	0.58 (0.10-1.80)	0.69 (0.12-2.16)	0.63 (0.10-1.96)
Pleural effusion	20 (2.1)	21605 (2.3)	0.90 (0.56-1.36)	1.08 (0.66-1.64)	0.99 (0.61-1.50)
Pneumothorax	<5	4006 (0.4)	1.22 (0.44-2.63)	1.13 (0.40-2.44)	1.11 (0.40-2.39)
Primary pulmonary hypertension	<5	1298 (0.1)	2.26 (0.56-5.88)	2.84 (0.70-7.41)	2.72 (0.67-7.12)
Pulmonary collapse (excl pneumothorax)	6 (0.6)	9986 (1.1)	0.58 (0.23-1.19)	0.67 (0.27-1.37)	0.60 (0.24-1.23)
Sleep apnoea	8 (0.8)	13482 (1.4)	0.58 (0.26-1.08)	0.55 (0.25-1.03)	0.53 (0.24-0.99)
**Renal CKD**					
CKD	81 (8.4)	83907 (8.9)	0.94 (0.74-1.17)	1.27 (0.98-1.62)	1.17 (0.91-1.49)
End stage renal disease	16 (1.7)	36953 (3.9)	0.41 (0.24-0.65)	0.48 (0.28-0.76)	0.46 (0.27-0.73)
**Renal Other**					
Acute Kidney Injury	15 (1.6)	33515 (3.6)	0.43 (0.25-0.69)	0.51 (0.29-0.82)	0.45 (0.26-0.73)
Glomerulonephritis	8 (0.8)	5240 (0.6)	1.49 (0.68-2.79)	1.65 (0.75-3.10)	1.59 (0.72-2.98)
Tubulo-interstitial nephritis	7 (0.7)	4847 (0.5)	1.41 (0.61-2.74)	1.63 (0.70-3.16)	1.49 (0.64-2.89)
**Endocrine Diabetes**					
Diabetic neurological complications	6 (0.6)	5000 (0.5)	1.17 (0.46-2.38)	1.33 (0.52-2.71)	1.12 (0.44-2.29)
T1DM	<5	3930 (0.4)	1.24 (0.44-2.68)	1.16 (0.42-2.50)	1.14 (0.41-2.46)
T2DM	50 (5.2)	57872 (6.1)	0.83 (0.62-1.10)	0.95 (0.70-1.25)	0.85 (0.63-1.13)
**Endocrine Obesity**					
Obesity	48 (5)	59076 (6.3)	0.78 (0.58-1.03)	0.89 (0.65-1.17)	0.80 (0.59-1.06)
**Endocrine Other**					
Polycystic ovarian syndrome	<5	7119 (0.8)	0.14 (0.01-0.60)	0.16 (0.01-0.69)	0.15 (0.01-0.68)
Syndrome of inappropriate secretion of antidiuretic hormone	<5	865 (0.1)	1.13 (0.06-4.98)	1.47 (0.08-6.50)	1.38 (0.08-6.14)
Thyroid disease	37 (3.8)	60429 (6.4)	0.58 (0.41-0.80)	0.76 (0.54-1.05)	0.75 (0.52-1.03)

HIV=human immunodeficiency virus. 95%CI=95% confidence interval. OR=odds ratio. IMD = index of multiple deprivation. COPD=chronic obstructive pulmonary disease. CKD=chronic kidney disease.

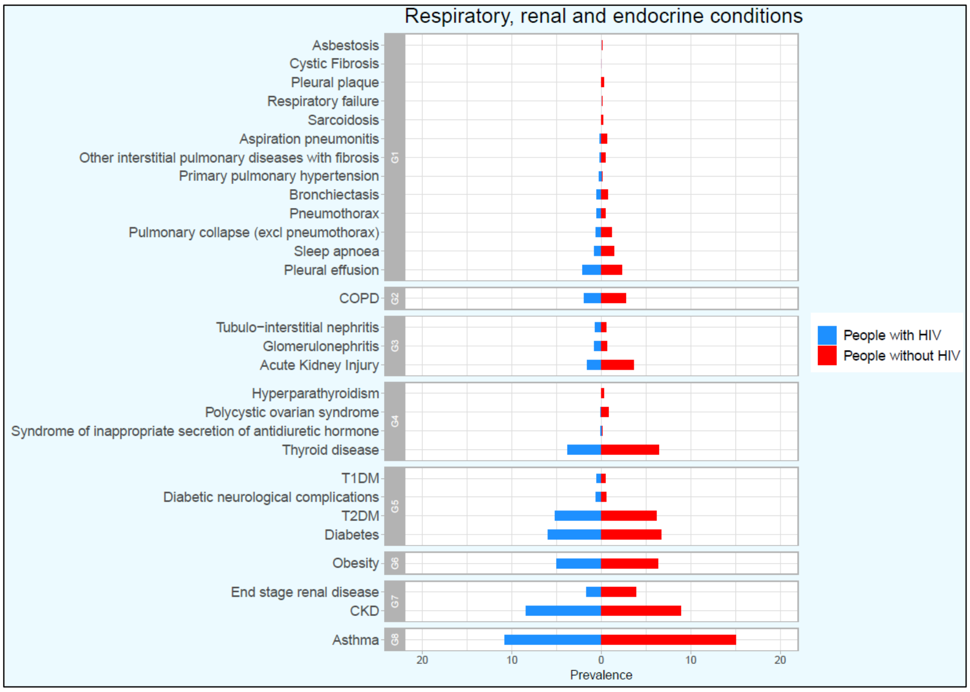

A.

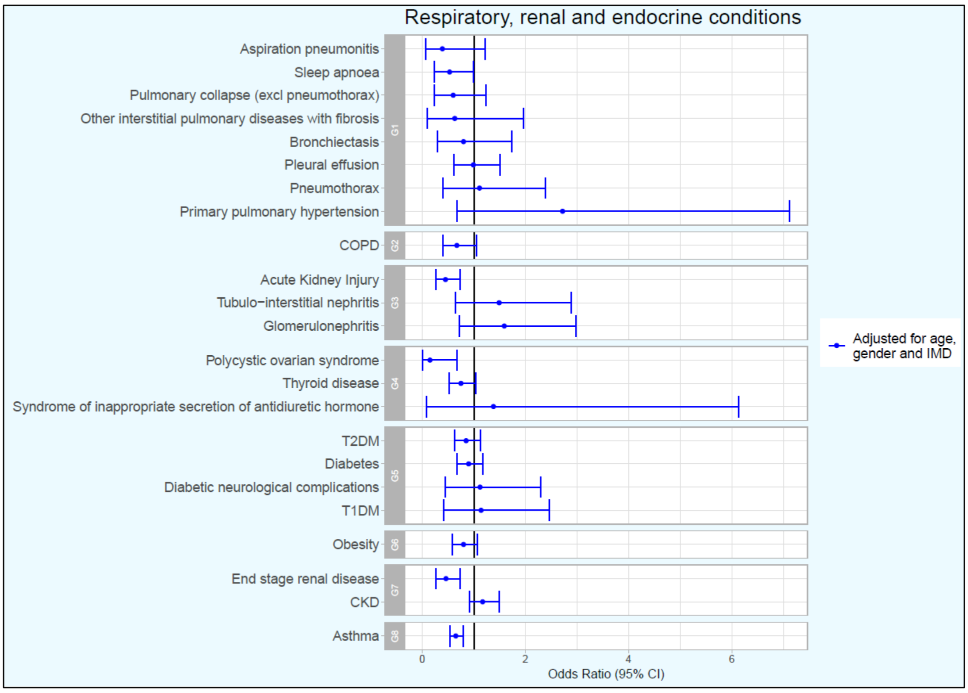

B.

Figure A4. Prevalence (A) and odds ratios (B) of individual respiratory, renal and endocrine conditions in adults with HIV compared to those without HIV.

HIV=human immunodeficiency virus. 95%CI=95% confidence interval. OR=odds ratio. IMD = index of multiple deprivation. COPD=chronic obstructive pulmonary disease. CKD=chronic kidney disease.

G1 = Respiratory_Other, G2 = Respiratory_COPD, G3 = Renal_Other, G4 = Endocrine_Other, G5 = Endocrine_Diabetes, G6 = Endocrine_Obesity, G7 = Renal_CKD, G8 = Respiratory_Asthma.

Table A7. Prevalence and odds ratios of individual GI, gynaecological and GU conditions in adults with HIV compared to those without HIV.

Health condition	People with HIV, n (%) (N=964)	People without HIV, n (%) (N=941113)	Unadjusted OR (95% CI)	Adjusted for age and sex OR (95% CI)	Adjusted for age, sex and IMD OR (95% CI)
**GI Disorder**					
Barrett's oesophagus	<5	8206 (0.9)	0.47 (0.15-1.10)	0.52 (0.16-1.21)	0.50 (0.15-1.16)
Coeliac disease	<5	4351 (0.5)	0.45 (0.07-1.38)	0.50 (0.08-1.56)	0.52 (0.09-1.60)
Crohn's disease	6 (0.6)	3836 (0.4)	1.53 (0.61-3.11)	1.58 (0.63-3.22)	1.59 (0.63-3.24)
Diverticular disease of intestine (acute and chronic)	9 (0.9)	33472 (3.6)	0.26 (0.12-0.46)	0.32 (0.15-0.58)	0.31 (0.15-0.57)
Gastritis and duodenitis	61 (6.3)	46323 (4.9)	1.30 (1.00-1.68)	1.42 (1.08-1.82)	1.32 (1.00-1.69)
Gastro-oesophageal reflux disease	80 (8.3)	91664 (9.7)	0.84 (0.66-1.05)	0.94 (0.74-1.17)	0.91 (0.72-1.14)
Irritable bowel syndrome	49 (5.1)	71248 (7.6)	0.65 (0.48-0.86)	0.75 (0.56-1.00)	0.77 (0.57-1.02)
Oesophagitis and oesophageal ulcer	81 (8.4)	68633 (7.3)	1.17 (0.92-1.45)	1.31 (1.03-1.64)	1.28 (1.00-1.60)
Pancreatitis	9 (0.9)	6052 (0.6)	1.46 (0.70-2.64)	1.62 (0.78-2.94)	1.47 (0.70-2.67)
Peptic ulcer disease	14 (1.5)	19502 (2.1)	0.70 (0.39-1.13)	0.77 (0.43-1.26)	0.71 (0.40-1.16)
Ulcerative colitis	10 (1)	6007 (0.6)	1.63 (0.82-2.87)	1.75 (0.87-3.08)	1.82 (0.91-3.22)
**GI Surgical**					
Anal fissure	52 (5.4)	27913 (3)	1.87 (1.39-2.44)	1.92 (1.44-2.51)	1.99 (1.49-2.61)
Anorectal fistula	27 (2.8)	6264 (0.7)	4.30 (2.86-6.18)	4.24 (2.82-6.09)	4.22 (2.81-6.07)
Anorectal prolapse	<5	3961 (0.4)	0.99 (0.31-2.30)	1.24 (0.38-2.91)	1.25 (0.39-2.93)
Appendicitis	67 (7)	68382 (7.3)	0.95 (0.74-1.21)	1.03 (0.79-1.31)	1.04 (0.80-1.33)
Cholecystitis	7 (0.7)	21113 (2.2)	0.32 (0.14-0.62)	0.39 (0.17-0.75)	0.36 (0.15-0.70)
Cholelithiasis	19 (2)	26434 (2.8)	0.70 (0.43-1.06)	0.88 (0.54-1.35)	0.85 (0.52-1.30)
**Gynaecological-GU Disorder**					
Dysmenorrhoea	31 (3.2)	36363 (3.9)	0.83 (0.57-1.16)	1.02 (0.69-1.45)	0.99 (0.67-1.40)
Endometrial hyperplasia and hypertrophy	<5	5243 (0.6)	0.56 (0.14-1.45)	0.78 (0.19-2.05)	0.75 (0.19-1.97)
Endometriosis	<5	10047 (1.1)	0.48 (0.17-1.04)	0.63 (0.23-1.37)	0.67 (0.24-1.46)
Erectile dysfunction	108 (11.2)	53186 (5.7)	2.11 (1.71-2.56)	2.05 (1.64-2.54)	1.98 (1.59-2.46)
Female genital prolapse	7 (0.7)	27628 (2.9)	0.24 (0.10-0.47)	0.36 (0.15-0.71)	0.37 (0.16-0.73)
Female infertility	28 (2.9)	16998 (1.8)	1.63 (1.09-2.32)	1.94 (1.30-2.78)	2.01 (1.34-2.88)
Female pelvic inflammatory disease	<5	2710 (0.3)	0.72 (0.12-2.23)	0.97 (0.16-3.00)	0.94 (0.16-2.94)
Hyperplasia of prostate	22 (2.3)	38807 (4.1)	0.54 (0.35-0.81)	0.51 (0.32-0.77)	0.51 (0.32-0.77)
Male infertility	29 (3)	14615 (1.6)	1.97 (1.33-2.79)	2.14 (1.44-3.03)	2.23 (1.50-3.16)
Menorrhagia and polymenorrhoea	74 (7.7)	88577 (9.4)	0.80 (0.63-1.01)	1.08 (0.84-1.39)	1.05 (0.81-1.35)
Neuromuscular dysfunction of bladder	9 (0.9)	10301 (1.1)	0.85 (0.41-1.54)	1.04 (0.50-1.89)	1.03 (0.49-1.87)
Non-acute cystitis	<5	2811 (0.3)	0.35 (0.02-1.53)	0.43 (0.02-1.91)	0.43 (0.02-1.90)
Obstructive and reflux uropathy	7 (0.7)	10863 (1.2)	0.63 (0.27-1.22)	0.66 (0.28-1.28)	0.63 (0.27-1.23)
Postmenopausal bleeding	9 (0.9)	23875 (2.5)	0.36 (0.17-0.66)	0.55 (0.26-1.01)	0.56 (0.27-1.02)
Urinary Incontinence	26 (2.7)	44020 (4.7)	0.56 (0.37-0.82)	0.77 (0.50-1.12)	0.73 (0.48-1.07)
Urolithiasis	42 (4.4)	37271 (4)	1.10 (0.80-1.48)	1.13 (0.82-1.53)	1.12 (0.81-1.51)

HIV=human immunodeficiency virus. 95%CI=95% confidence interval. OR=odds ratio. IMD = index of multiple deprivation.

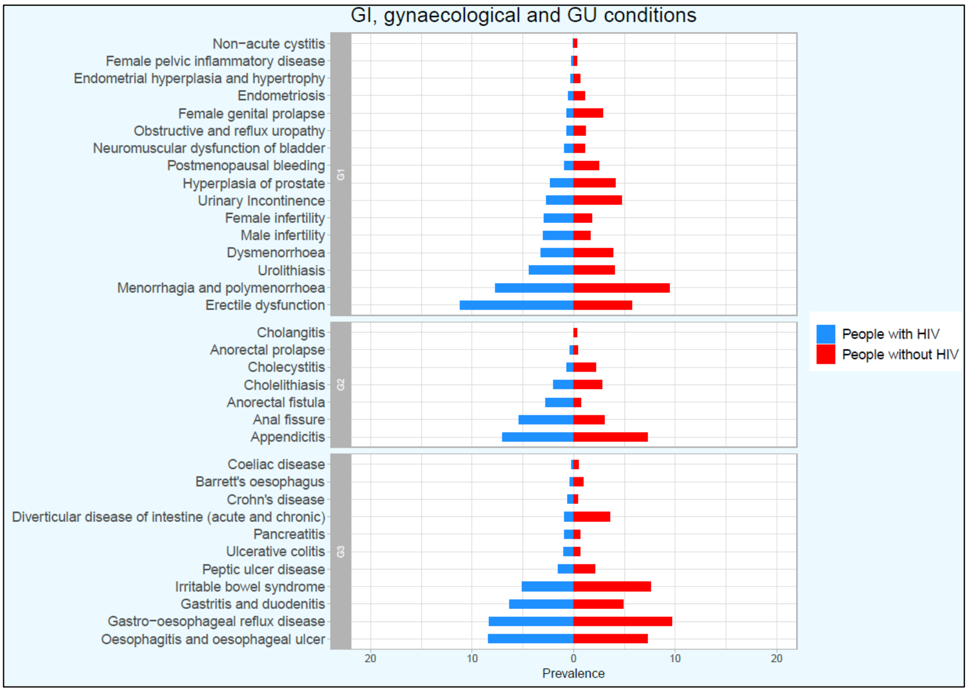

A.

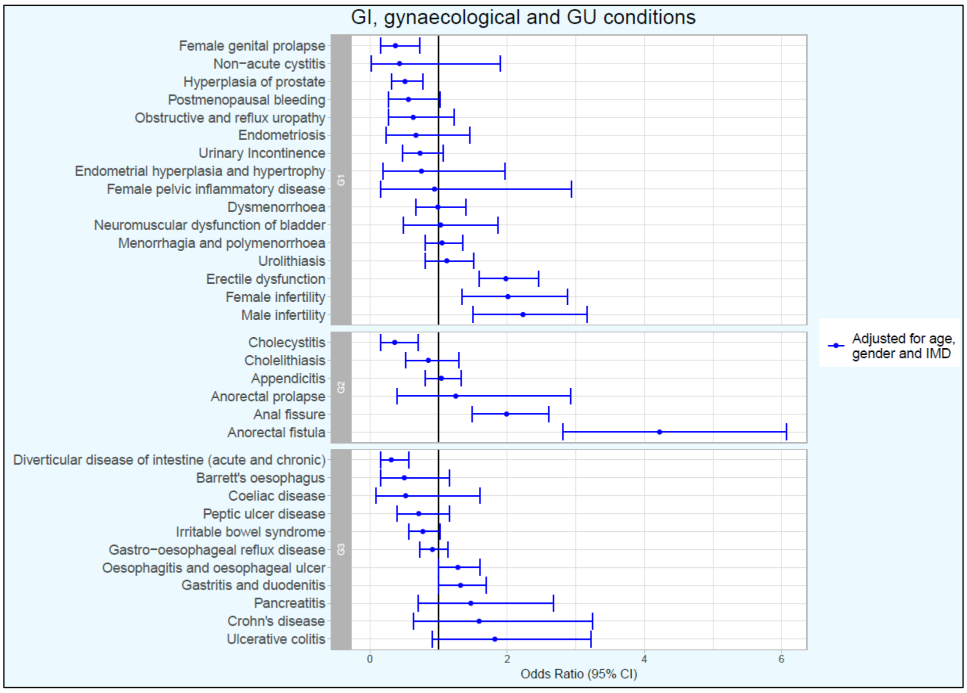

B.

Figure A5. Prevalence and odds ratios of individual GI, gynaecological and GU conditions in adults with HIV compared to those without HIV.

HIV=human immunodeficiency virus. 95%CI=95% confidence interval. OR=odds ratio. IMD = index of multiple deprivation.

G1 = Gu Disorder, G2 = GI_Surgical, G3 = Gi Disorder.

Table A8. Prevalence and odds ratios of individual conditions related to the special senses in adults with HIV compared to those without HIV.

Health condition	People with HIV, n (%) (N=964)	People without HIV, n (%) (N=941113)	Unadjusted OR (95% CI)	Adjusted for age and sex OR (95% CI)	Adjusted for age, sex and IMD OR (95% CI)
**Ear, Nose and Throat**					
Allergic and chronic rhinitis	153 (15.9)	165524 (17.6)	0.88 (0.74-1.05)	0.88 (0.74-1.04)	0.91 (0.76-1.07)
Chronic sinusitis	44 (4.6)	64051 (6.8)	0.65 (0.48-0.87)	0.75 (0.54-1.00)	0.77 (0.56-1.03)
Hearing loss	47 (4.9)	90395 (9.6)	0.48 (0.35-0.64)	0.52 (0.38-0.69)	0.51 (0.38-0.68)
Hypertrophy of nasal turbinates	8 (0.8)	4805 (0.5)	1.63 (0.74-3.05)	1.50 (0.68-2.81)	1.47 (0.67-2.75)
Keratitis	10 (1)	5477 (0.6)	1.79 (0.89-3.15)	1.96 (0.98-3.45)	2.05 (1.02-3.62)
Meniere disease	<5	4070 (0.4)	0.24 (0.01-1.05)	0.30 (0.02-1.33)	0.30 (0.02-1.30)
Nasal polyp	7 (0.7)	10910 (1.2)	0.62 (0.27-1.21)	0.64 (0.28-1.25)	0.66 (0.28-1.29)
**Eye**					
Anterior and Intermediate Uveitis	<5	7870 (0.8)	0.62 (0.22-1.33)	0.67 (0.24-1.45)	0.68 (0.24-1.47)
Blindness	9 (0.9)	9011 (1)	0.97 (0.47-1.76)	1.15 (0.55-2.09)	1.07 (0.51-1.94)
Cataract	34 (3.5)	75210 (8)	0.42 (0.29-0.58)	0.52 (0.36-0.73)	0.50 (0.34-0.70)
Diabetic ophthalmic complications	15 (1.6)	21664 (2.3)	0.67 (0.38-1.08)	0.75 (0.43-1.21)	0.69 (0.40-1.12)
Eye infections	6 (0.6)	692 (0.1)	8.51 (3.37-17.38)	9.21 (3.64-18.82)	8.61 (3.40-17.61)
Glaucoma	11 (1.1)	29204 (3.1)	0.36 (0.19-0.62)	0.40 (0.21-0.69)	0.40 (0.20-0.69)
Keratitis	10 (1)	5477 (0.6)	1.79 (0.89-3.15)	1.96 (0.98-3.45)	2.05 (1.02-3.62)
Macular degeneration	<5	16561 (1.8)	0.23 (0.07-0.54)	0.31 (0.10-0.73)	0.30 (0.09-0.70)
Posterior Uveitis	10 (1)	635 (0.1)	15.52 (7.73-27.50)	16.97 (8.44-30.10)	16.83 (8.36-29.88)
Ptosis of eyelid	10 (1)	7508 (0.8)	1.30 (0.65-2.30)	1.53 (0.76-2.71)	1.51 (0.76-2.68)
Retinal detachments and breaks	12 (1.2)	7146 (0.8)	1.65 (0.88-2.78)	1.82 (0.97-3.08)	1.89 (1.01-3.19)
Retinal vascular occlusions	<5	3670 (0.4)	0.27 (0.02-1.17)	0.33 (0.02-1.45)	0.33 (0.02-1.44)
Scleritis and episcleritis	7 (0.7)	5329 (0.6)	1.28 (0.55-2.49)	1.46 (0.63-2.84)	1.56 (0.67-3.03)
**Skin**					
Acne	89 (9.2)	84949 (9)	1.03 (0.82-1.27)	0.93 (0.74-1.15)	0.96 (0.76-1.20)
Actinic keratosis	16 (1.7)	29879 (3.2)	0.51 (0.30-0.81)	0.60 (0.35-0.96)	0.66 (0.39-1.06)
Alopecia areata	<5	4632 (0.5)	0.84 (0.26-1.96)	0.85 (0.26-1.99)	0.85 (0.26-1.98)
Dermatitis (atopc/contact/other/unspecified)	230 (23.9)	246160 (26.2)	0.88 (0.76-1.02)	0.92 (0.79-1.07)	0.93 (0.80-1.08)
Hidradenitis suppurativa	7 (0.7)	3800 (0.4)	1.80 (0.77-3.50)	1.96 (0.84-3.82)	1.83 (0.78-3.55)
Lichen planus	7 (0.7)	6904 (0.7)	0.99 (0.42-1.92)	1.17 (0.50-2.27)	1.20 (0.51-2.33)
Psoriasis	39 (4)	40604 (4.3)	0.94 (0.67-1.27)	0.98 (0.70-1.33)	0.97 (0.69-1.32)
Rosacea	24 (2.5)	31168 (3.3)	0.75 (0.48-1.09)	0.84 (0.54-1.23)	0.87 (0.56-1.28)
Seborrheic dermatitis	67 (7)	50888 (5.4)	1.31 (1.01-1.66)	1.36 (1.05-1.73)	1.37 (1.06-1.74)
Urticaria	54 (5.6)	54154 (5.8)	0.97 (0.73-1.27)	1.03 (0.77-1.34)	1.03 (0.77-1.34)
Vitiligo	<5	4121 (0.4)	0.95 (0.29-2.21)	0.97 (0.30-2.26)	1.00 (0.31-2.34)

HIV=human immunodeficiency virus. 95%CI=95% confidence interval. OR=odds ratio. IMD = index of multiple deprivation.

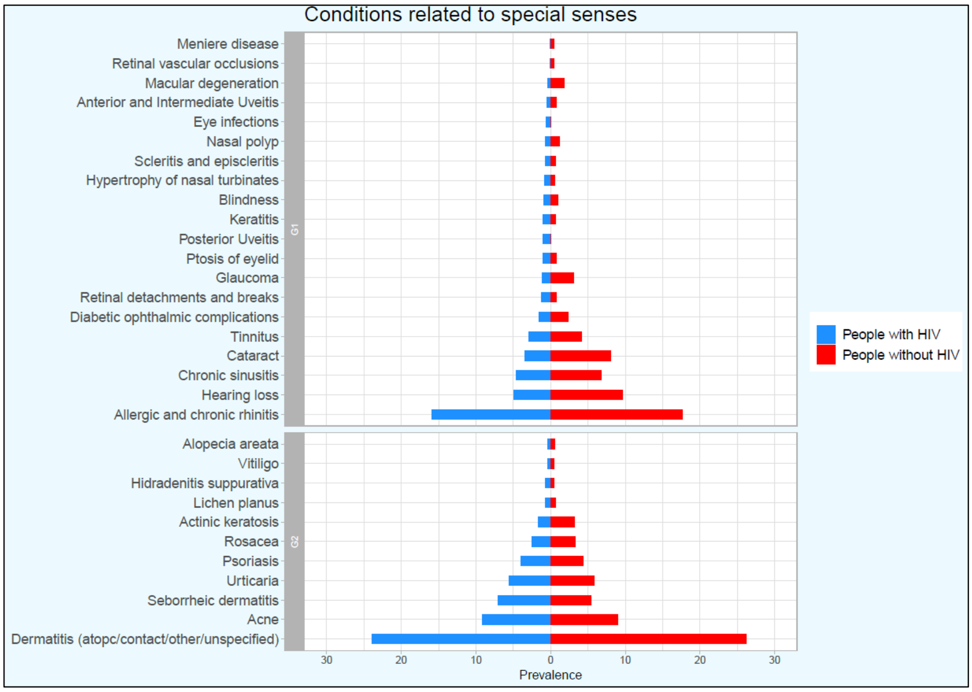

A.

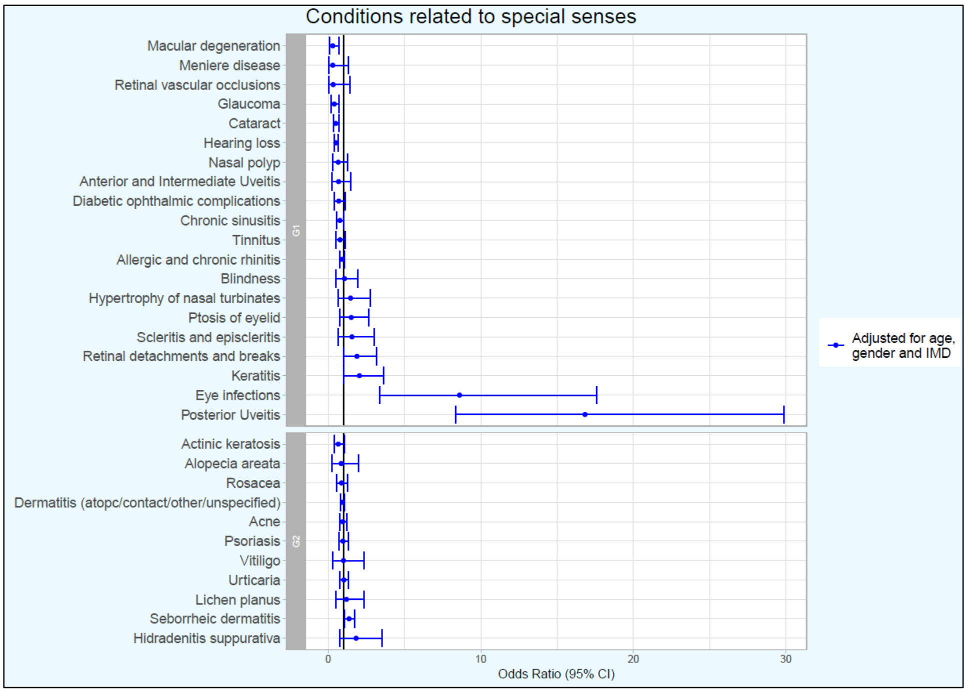

B.

Figure A6. Prevalence and odds ratios of individual conditions related to the special senses in adults with HIV compared to those without HIV.

HIV=human immunodeficiency virus. 95%CI=95% confidence interval. OR=odds ratio. IMD = index of multiple deprivation.

G1 = Eye_ENT Disorder, G2 = Dermatology.

Table A9. Prevalence and odds ratios of individual CNS-related conditions in adults with HIV compared to those without HIV.

Health condition	People with HIV, n (%) (N=964)	People without HIV, n (%) (N=941113)	Unadjusted OR (95% CI)	Adjusted for age and sex OR (95% CI)	Adjusted for age, sex and IMD OR (95% CI)
**CNS Neurological**					
Disorders of autonomic nervous system	<5	2560 (0.3)	0.76 (0.13-2.36)	0.86 (0.14-2.68)	0.81 (0.13-2.50)
Epilepsy	20 (2.1)	15491 (1.6)	1.27 (0.79-1.92)	1.27 (0.79-1.92)	1.17 (0.72-1.76)
Migraine	60 (6.2)	83581 (8.9)	0.68 (0.52-0.88)	0.76 (0.58-0.98)	0.76 (0.58-0.98)
Parkinson's disease	<5	4032 (0.4)	0.48 (0.08-1.50)	0.58 (0.10-1.80)	0.57 (0.09-1.77)
**CNS Peripheral**					
Bell's palsy	27 (2.8)	8867 (0.9)	3.03 (2.02-4.35)	3.24 (2.16-4.66)	3.08 (2.04-4.42)
Peripheral neuropathies (excluding cranial nerve and carpal tunnel syndromes)	36 (3.7)	21665 (2.3)	1.65 (1.16-2.26)	1.84 (1.29-2.54)	1.79 (1.25-2.46)
Trigeminal neuralgia	<5	6634 (0.7)	0.59 (0.18-1.37)	0.73 (0.23-1.71)	0.73 (0.23-1.70)
**CNS Haematological**					
Intracerebral haemorrhage	<5	1528 (0.2)	0.64 (0.04-2.82)	0.74 (0.04-3.27)	0.69 (0.04-3.07)
Subdural haematoma - nontraumatic	<5	1217 (0.1)	1.61 (0.27-4.97)	1.86 (0.31-5.79)	1.74 (0.29-5.40)
**CNS Other**					
Essential tremor	<5	3214 (0.3)	0.30 (0.02-1.34)	0.35 (0.02-1.53)	0.35 (0.02-1.53)
**CNS Psychiatry**					
Alcohol Problems	71 (7.4)	37330 (4)	1.92 (1.50-2.43)	1.85 (1.44-2.34)	1.64 (1.27-2.07)
Anxiety disorders	151 (15.7)	147314 (15.7)	1.00 (0.84-1.19)	1.11 (0.93-1.32)	1.06 (0.88-1.25)
Autism and Asperger's syndrome	<5	3084 (0.3)	0.95 (0.24-2.47)	0.71 (0.18-1.85)	0.68 (0.17-1.78)
Bipolar affective disorder and mania	11 (1.1)	3658 (0.4)	2.96 (1.53-5.09)	3.19 (1.65-5.49)	2.93 (1.52-5.05)
Dementia	9 (0.9)	16674 (1.8)	0.52 (0.25-0.95)	0.73 (0.35-1.34)	0.67 (0.32-1.22)
Depression	261 (27.1)	194874 (20.7)	1.42 (1.23-1.64)	1.61 (1.39-1.86)	1.49 (1.28-1.71)
Eating disorders	<5	3728 (0.4)	1.05 (0.32-2.44)	1.24 (0.38-2.91)	1.30 (0.40-3.05)
Hyperkinetic disorders	<5	2625 (0.3)	1.12 (0.28-2.90)	0.79 (0.20-2.06)	0.72 (0.18-1.88)
Intellectual disability	<5	6717 (0.7)	0.58 (0.18-1.35)	0.52 (0.16-1.20)	0.44 (0.14-1.03)
Obsessive-compulsive disorder	<5	5092 (0.5)	0.38 (0.06-1.18)	0.38 (0.06-1.17)	0.38 (0.06-1.17)
Personality disorders	8 (0.8)	5926 (0.6)	1.32 (0.60-2.47)	1.33 (0.60-2.48)	1.12 (0.51-2.10)
Schizophrenia, schizotypal and delusional disorders	12 (1.2)	6685 (0.7)	1.76 (0.94-2.97)	1.73 (0.92-2.92)	1.42 (0.76-2.39)
Substance misuse	50 (5.2)	15215 (1.6)	3.33 (2.47-4.38)	2.97 (2.20-3.91)	2.45 (1.81-3.23)

HIV=human immunodeficiency virus. 95%CI=95% confidence interval. OR=odds ratio. IMD = index of multiple deprivation.

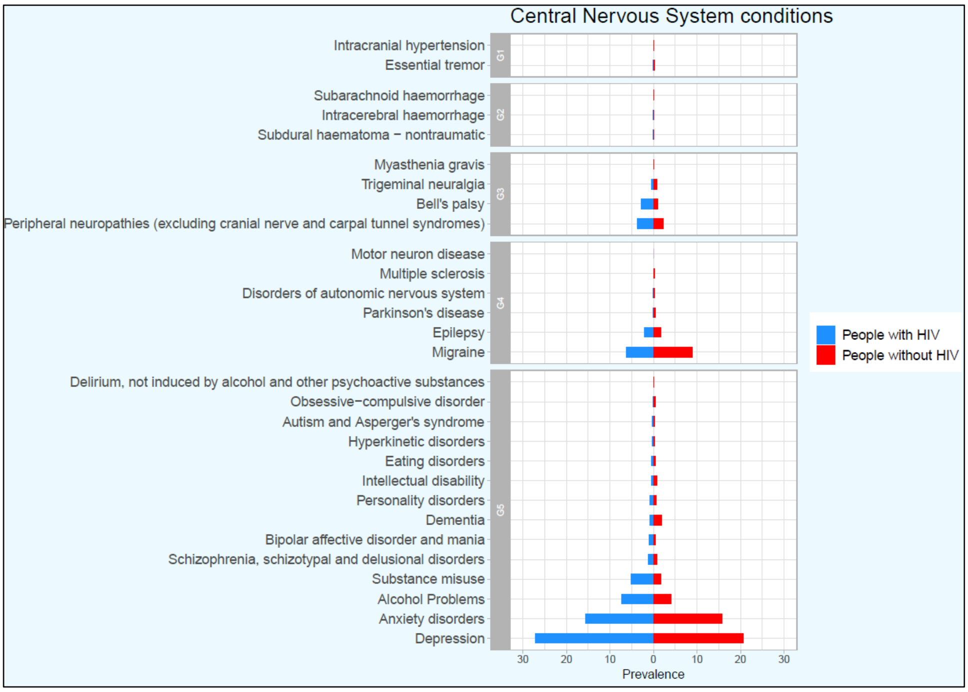

A.

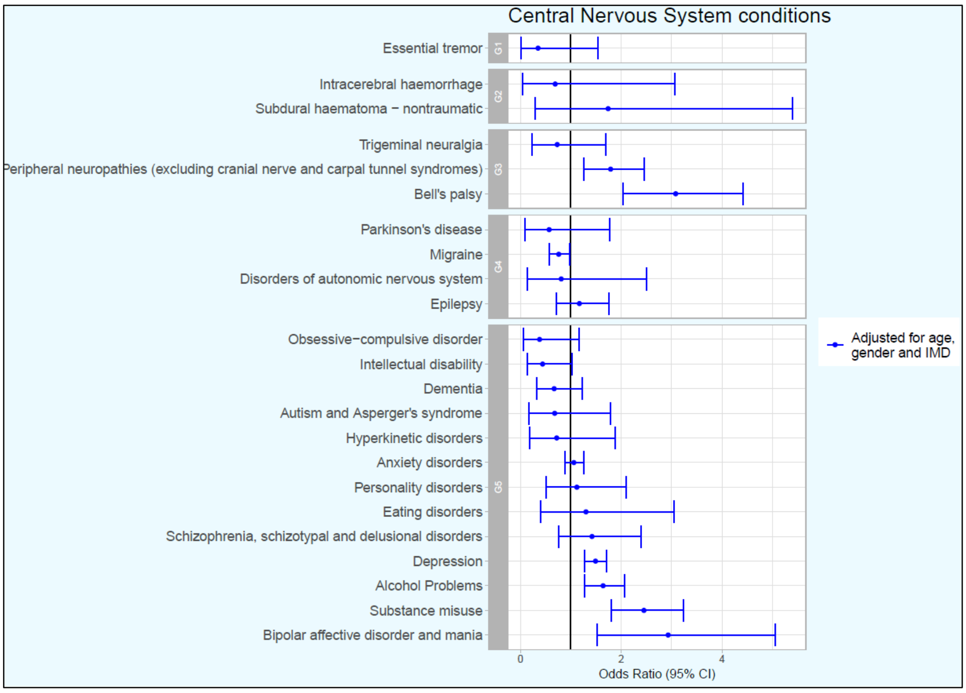

B.

Figure A7. Prevalence and odds ratios of individual CNS-related conditions in adults with HIV compared to those without HIV.

HIV=human immunodeficiency virus. 95%CI=95% confidence interval. OR=odds ratio. IMD = index of multiple deprivation.

G1 = CNS_Other, G2 = CNS_Haem, G3 = CNS_Peripheral, G4 = CNS_Neuro, G5 = CNS_Psychiatry.

Table A10. Prevalence and odds ratios of individual benign neoplasms and malignant conditions in adults with HIV compared to those without HIV.

Health condition	People with HIV, n (%) (N=964)	People without HIV, n (%) (N=941113)	Unadjusted OR (95% CI)	Adjusted for age and sex OR (95% CI)	Adjusted for age, sex and IMD OR (95% CI)
**Benign Neoplasms**					
Benign neoplasm and polyp of uterus	<5	12627 (1.3)	0.31 (0.09-0.71)	0.44 (0.14-1.03)	0.45 (0.14-1.04)
Benign neoplasm of brain and other parts of central nervous system	<5	2552 (0.3)	0.76 (0.13-2.37)	0.91 (0.15-2.81)	0.92 (0.15-2.86)
Benign neoplasm of colon, rectum, anus and anal canal	31 (3.2)	35617 (3.8)	0.84 (0.58-1.19)	0.94 (0.64-1.33)	0.93 (0.63-1.31)
Benign neoplasm of ovary	20 (2.1)	26131 (2.8)	0.74 (0.46-1.12)	0.99 (0.61-1.51)	0.98 (0.60-1.50)
Benign neoplasm of stomach and duodenum	<5	9607 (1)	0.51 (0.18-1.09)	0.62 (0.22-1.35)	0.62 (0.22-1.33)
Cervical Intra-epithelial Neoplasia	34 (3.5)	9359 (1)	3.64 (2.53-5.04)	4.94 (3.40-6.92)	4.75 (3.28-6.66)
**Malignancy Breast**					
Primary Malignancy_Breast	<5	13999 (1.5)	0.35 (0.12-0.74)	0.53 (0.19-1.14)	0.54 (0.19-1.18)
**Malignancy GI**					
Primary Malignancy_colorectal and anus	10 (1)	6550 (0.7)	1.50 (0.75-2.63)	1.80 (0.90-3.20)	1.78 (0.88-3.15)
Primary Malignancy_Oesophageal	<5	459 (0)	4.26 (0.71-13.24)	4.80 (0.80-14.99)	4.57 (0.76-14.26)
Primary Malignancy_Oro-pharyngeal	<5	1344 (0.1)	2.91 (0.90-6.80)	3.15 (0.97-7.37)	2.95 (0.91-6.92)
Primary Malignancy_Pancreatic	<5	157 (0)	6.22 (0.35-27.73)	7.68 (0.44-34.33)	7.51 (0.43-33.64)
Secondary malignancy_Liver_Bile duct	<5	3966 (0.4)	0.25 (0.01-1.08)	0.29 (0.02-1.27)	0.27 (0.02-1.21)
**Malignancy GU**					
Primary Malignancy_Cervical	<5	822 (0.1)	3.57 (0.89-9.31)	5.12 (1.27-13.42)	4.64 (1.15-12.15)
Primary Malignancy_Kidney and Ureter	<5	2139 (0.2)	0.91 (0.15-2.83)	1.03 (0.17-3.20)	1.01 (0.17-3.13)
Primary Malignancy_Prostate	7 (0.7)	11539 (1.2)	0.59 (0.25-1.14)	0.58 (0.25-1.13)	0.59 (0.25-1.17)
Primary Malignancy_Testicular	<5	819 (0.1)	1.19 (0.07-5.26)	0.98 (0.06-4.32)	1.02 (0.06-4.50)
**Malignancy Haem**					
Hodgkin Lymphoma	<5	672 (0.1)	4.37 (1.08-11.39)	4.46 (1.10-11.63)	4.66 (1.15-12.16)
Leukaemia	<5	1633 (0.2)	1.20 (0.20-3.70)	1.33 (0.22-4.11)	1.34 (0.22-4.15)
Non-Hodgkin Lymphoma	22 (2.3)	2212 (0.2)	9.91 (6.29-14.77)	11.56 (7.30-17.30)	11.92 (7.53-17.86)
Polycythaemia vera	<5	1187 (0.1)	1.65 (0.27-5.10)	1.73 (0.29-5.36)	1.61 (0.27-4.99)
**Malignancy Respiratory**					
Primary Malignancy_Lung and trachea	<5	1188 (0.1)	2.47 (0.61-6.43)	3.04 (0.75-7.96)	2.65 (0.66-6.94)
Secondary Malignancy_Lung	<5	2972 (0.3)	0.66 (0.11-2.03)	0.77 (0.13-2.39)	0.73 (0.12-2.27)
Secondary Malignancy_Pleura	<5	781 (0.1)	1.25 (0.07-5.52)	1.54 (0.09-6.80)	1.48 (0.08-6.56)
**Malignancy Other**					
Primary Malignancy_Bone and articular cartilage	<5	212 (0)	4.61 (0.26-20.47)	4.59 (0.26-20.43)	4.61 (0.26-20.51)
Primary Malignancy_Brain, Other CNS and Intracranial	<5	438 (0)	4.46 (0.74-13.87)	4.49 (0.74-13.97)	4.53 (0.75-14.10)
Primary Malignancy_Malignant Melanoma	<5	5405 (0.6)	0.36 (0.06-1.11)	0.43 (0.07-1.34)	0.47 (0.08-1.47)
Primary Malignancy_Other Organs	<5	3379 (0.4)	1.45 (0.52-3.12)	1.71 (0.61-3.69)	1.63 (0.58-3.54)
Primary Malignancy_Other Skin and subcutaneous tissue	20 (2.1)	28156 (3)	0.69 (0.43-1.04)	0.84 (0.51-1.28)	0.89 (0.55-1.36)
Secondary Malignancy_Bone	<5	3841 (0.4)	0.51 (0.08-1.57)	0.59 (0.10-1.82)	0.56 (0.09-1.74)
Secondary Malignancy_Other organs	<5	3541 (0.4)	0.27 (0.02-1.21)	0.32 (0.02-1.43)	0.32 (0.02-1.40)
Secondary Malignancy_retroperitoneum and peritoneum	<5	1739 (0.2)	0.56 (0.03-2.47)	0.70 (0.04-3.09)	0.69 (0.04-3.05)

HIV=human immunodeficiency virus. 95%CI=95% confidence interval. OR=odds ratio. IMD = index of multiple deprivation.

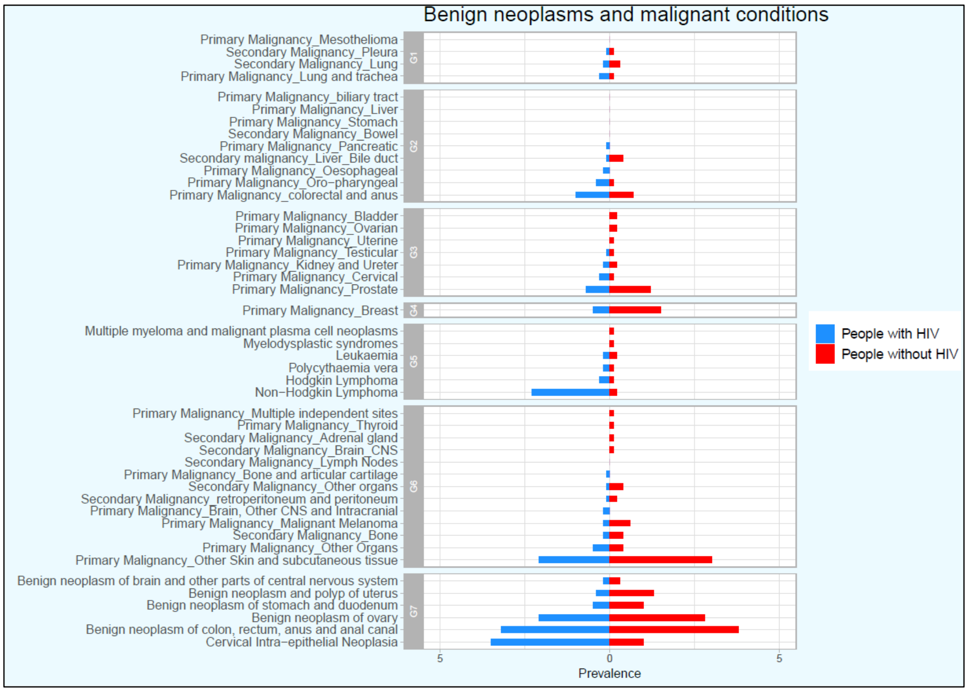

A.

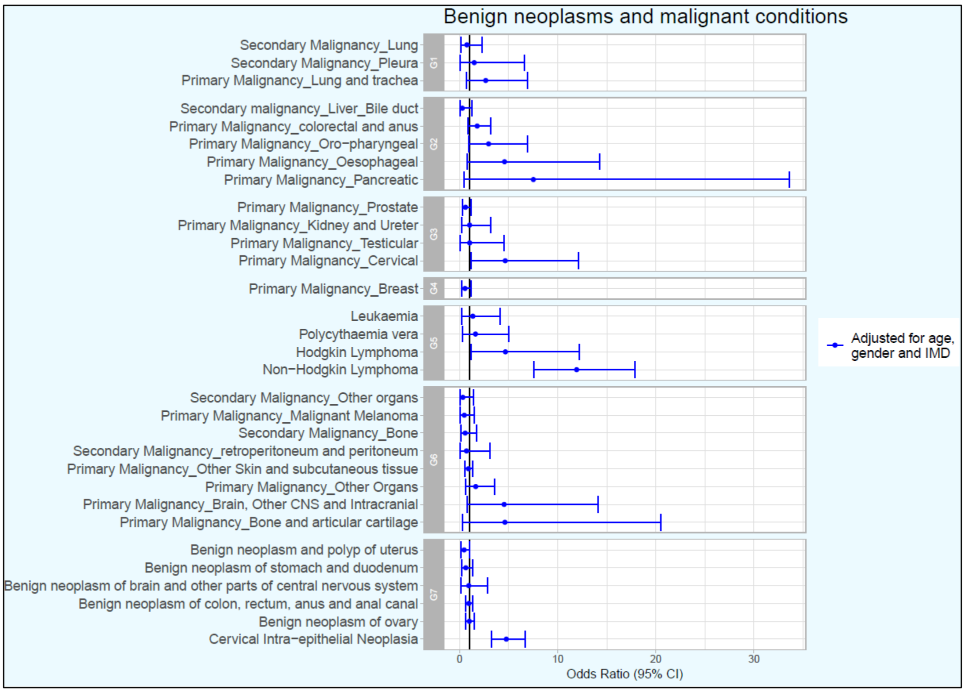

B.

Figure A8. Prevalence and odds ratios of individual benign neoplasms and malignant conditions in adults with HIV compared to those without HIV.

HIV=human immunodeficiency virus. 95%CI=95% confidence interval. OR=odds ratio. IMD = index of multiple deprivation.

Table A11. Prevalence and odds ratios of individual haematological conditions in adults with HIV compared to those without HIV.

Health condition	People with HIV, n (%) (N=964)	People without HIV, n (%) (N=941113)	Unadjusted OR (95% CI)	Adjusted for age and sex OR (95% CI)	Adjusted for age, sex and IMD OR (95% CI)
**Acquired**					
Agranulocytosis	27 (2.8)	7131 (0.8)	3.77 (2.51-5.42)	4.30 (2.85-6.18)	4.31 (2.86-6.20)
Aplastic anaemias	6 (0.6)	390 (0)	15.11 (5.96-30.95)	16.99 (6.70-34.87)	16.70 (6.58-34.34)
Folate deficiency anaemia	12 (1.2)	4421 (0.5)	2.67 (1.42-4.50)	3.18 (1.70-5.37)	2.65 (1.41-4.49)
Hyposplenism	<5	1697 (0.2)	1.15 (0.19-3.56)	1.21 (0.20-3.75)	1.18 (0.20-3.65)
Iron deficiency anaemia	40 (4.1)	35663 (3.8)	1.10 (0.79-1.49)	1.39 (0.99-1.89)	1.30 (0.93-1.77)
Other anaemias	61 (6.3)	43665 (4.6)	1.39 (1.06-1.78)	1.79 (1.36-2.31)	1.66 (1.26-2.14)
Other haemolytic anaemias	<5	838 (0.1)	4.68 (1.45-10.92)	4.82 (1.49-11.26)	4.82 (1.49-11.28)
Primary or Idiopathic Thrombocytopaenia	7 (0.7)	1778 (0.2)	3.86 (1.66-7.51)	4.19 (1.79-8.15)	4.26 (1.82-8.28)
Secondary or other Thrombocytopaenia	23 (2.4)	5684 (0.6)	4.02 (2.58-5.94)	4.49 (2.88-6.64)	4.30 (2.75-6.36)
Secondary polycythaemia	<5	1441 (0.2)	0.68 (0.04-2.99)	0.70 (0.04-3.08)	0.63 (0.04-2.78)
Splenomegaly	<5	2114 (0.2)	2.32 (0.83-5.00)	2.32 (0.83-5.02)	2.17 (0.78-4.69)
Vitamin B12 deficiency anaemia	14 (1.5)	16677 (1.8)	0.82 (0.46-1.33)	0.99 (0.56-1.62)	0.90 (0.50-1.47)
**Inherited**					
Sickle-cell anaemia	<5	371 (0)	7.92 (1.96-20.70)	8.14 (2.02-21.31)	6.49 (1.60-17.00)
Sickle-cell trait	14 (1.5)	1537 (0.2)	9.01 (5.05-14.69)	9.50 (5.32-15.52)	7.55 (4.22-12.35)
Thalassaemia	<5	1111 (0.1)	0.88 (0.05-3.88)	0.94 (0.05-4.14)	0.89 (0.05-3.92)
Thalassaemia trait	<5	1986 (0.2)	2.47 (0.88-5.32)	2.59 (0.93-5.60)	2.48 (0.89-5.36)
Thrombophilia	<5	1872 (0.2)	1.57 (0.39-4.08)	1.77 (0.44-4.61)	1.83 (0.45-4.77)

HIV=human immunodeficiency virus. 95%CI=95% confidence interval. OR=odds ratio. IMD = index of multiple deprivation.

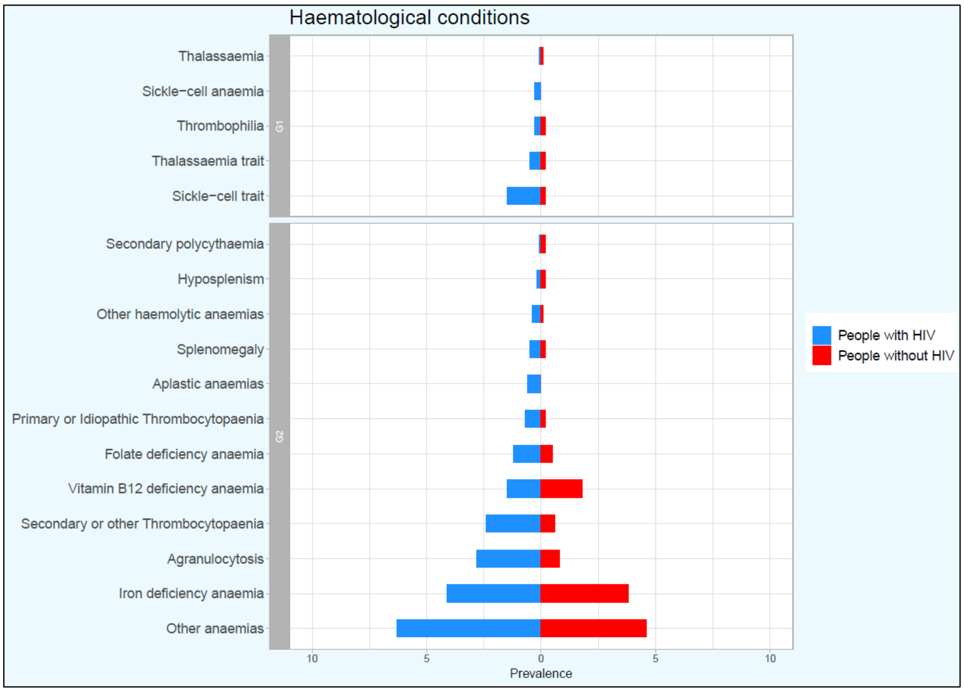

A.

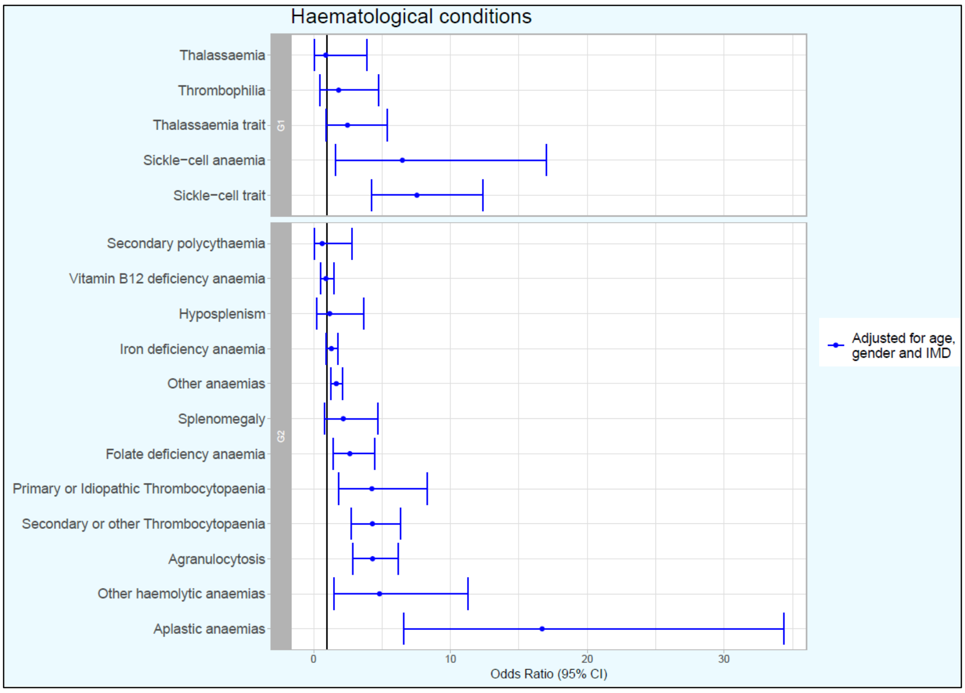

B.

Figure A9. Prevalence and odds ratios of individual haematological conditions in adults with HIV compared to those without HIV.

HIV=human immunodeficiency virus. 95%CI=95% confidence interval. OR=odds ratio. IMD = index of multiple deprivation.

G1 = Malignancy_Respiratory, G2 = Malignancy_GI, G3 = Malignancy_GU, G4 = Malignancy_Breast, G5 = Malignancy_Haem, G6 = Malignancy_Other, G7 = Beningn Neoplasms.

Table A12. Prevalence and odds ratios of individual infections in adults with HIV compared to those without HIV.

Health condition	People with HIV, n (%) (N=964)	People without HIV, n (%) (N=941113)	Unadjusted OR (95% CI)	Adjusted for age and sex OR (95% CI)	Adjusted for age, sex and IMD OR (95% CI)
**Infection_Bacterial**					
Bacterial Diseases (excl TB)	55 (5.7)	62884 (6.7)	0.85 (0.64-1.10)	1.01 (0.76-1.32)	0.93 (0.70-1.21)
**Infection_Viral**					
Viral diseases (excl chronic hepatitis/HIV)	50 (5.2)	6898 (0.7)	7.41 (5.50-9.75)	7.68 (5.70-10.11)	7.20 (5.34-9.48)
**Infection_Mycoses**					
Mycoses	47 (4.9)	432 (0)	111.61 (81.02-150.20)	120.86 (87.39-163.44)	114.49 (82.61-155.17)
**Infection_TB**					
Tuberculosis	67 (7)	6147 (0.7)	11.36 (8.77-14.46)	13.55 (10.42-17.33)	12.43 (9.56-15.90)
**Infection_CNS**					
Meningitis	<5	257 (0)	7.61 (1.26-23.73)	7.11 (1.18-22.21)	7.10 (1.17-22.20)
Other nervous system infections	<5	1294 (0.1)	3.79 (1.35-8.18)	4.21 (1.50-9.10)	3.97 (1.42-8.60)
**Infection_CVS**					
Infections of the Heart	<5	740 (0.1)	1.32 (0.08-5.83)	1.29 (0.07-5.72)	1.26 (0.07-5.56)
Rheumatic fever	<5	2171 (0.2)	0.45 (0.03-1.98)	0.56 (0.03-2.49)	0.56 (0.03-2.47)
**Infection_GI**					
Chronic viral hepatitis	47 (4.9)	1804 (0.2)	26.69 (19.57-35.48)	25.99 (19.05-34.57)	22.17 (16.23-29.53)
Infection of anal and rectal regions	18 (1.9)	556 (0.1)	32.19 (19.32-50.10)	35.87 (21.50-55.96)	32.39 (19.39-50.61)
Infection of liver	<5	473 (0.1)	2.07 (0.12-9.13)	2.26 (0.13-10.02)	2.03 (0.12-9.00)
Infections of the digestive system	12 (1.2)	4070 (0.4)	2.90 (1.55-4.89)	3.09 (1.65-5.22)	2.66 (1.42-4.50)
Peritonitis	<5	9467 (1)	0.41 (0.13-0.96)	0.45 (0.14-1.05)	0.43 (0.13-1.00)
**Infection_GU**					
Infection of other or unspecified genitourinary system	7 (0.7)	3192 (0.3)	2.15 (0.92-4.17)	2.62 (1.12-5.12)	2.33 (0.99-4.54)
Urinary Tract Infections	20 (2.1)	49211 (5.2)	0.38 (0.24-0.58)	0.47 (0.29-0.72)	0.43 (0.27-0.66)
**Infection_Respiratory**					
Ear and Upper Respiratory Tract Infections	<5	3985 (0.4)	0.73 (0.18-1.91)	0.69 (0.17-1.79)	0.65 (0.16-1.69)
Lower Respiratory Tract Infections	63 (6.5)	40323 (4.3)	1.56 (1.20-2.00)	1.90 (1.45-2.45)	1.65 (1.26-2.13)
**Infection_Skin**					
Infection of skin and subcutaneous tissues	8 (0.8)	4628 (0.5)	1.69 (0.77-3.17)	1.56 (0.71-2.92)	1.45 (0.66-2.70)
**Infection_Other**					
Infection of bones and joints	<5	621 (0.1)	4.73 (1.17-12.33)	5.04 (1.25-13.18)	4.61 (1.14-12.05)
Infections of Other or unspecified organs	194 (20.1)	12729 (1.4)	18.38 (15.64-21.48)	21.98 (18.64-25.81)	20.14 (17.06-23.65)
Other or unspecified infectious organisms	88 (9.1)	73397 (7.8)	1.19 (0.95-1.47)	1.34 (1.06-1.66)	1.20 (0.96-1.49)
Parasitic infections	11 (1.1)	651 (0.1)	16.67 (8.60-28.84)	16.73 (8.62-28.96)	14.98 (7.72-25.96)
Septicaemia	<5	269 (0)	3.63 (0.21-16.11)	3.48 (0.20-15.46)	3.32 (0.19-14.77)

HIV=human immunodeficiency virus. 95%CI=95% confidence interval. OR=odds ratio. IMD = index of multiple deprivation. TB=tuberculosis. CNS=central nervous system. CVS=cardiovascular. GI=gastrointestinal. GU=genitourinary.

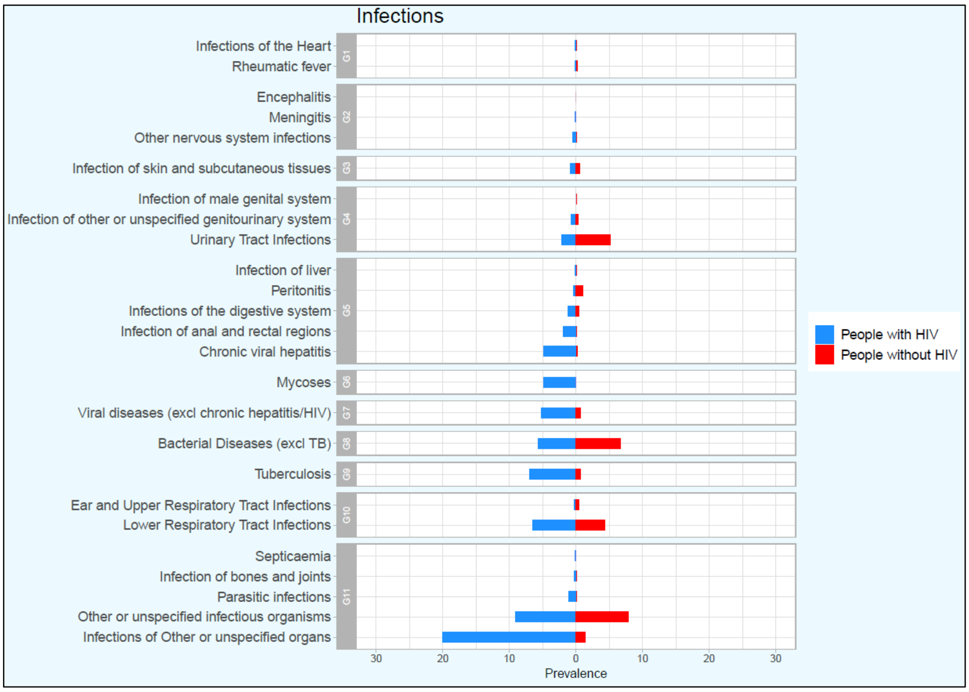

A.

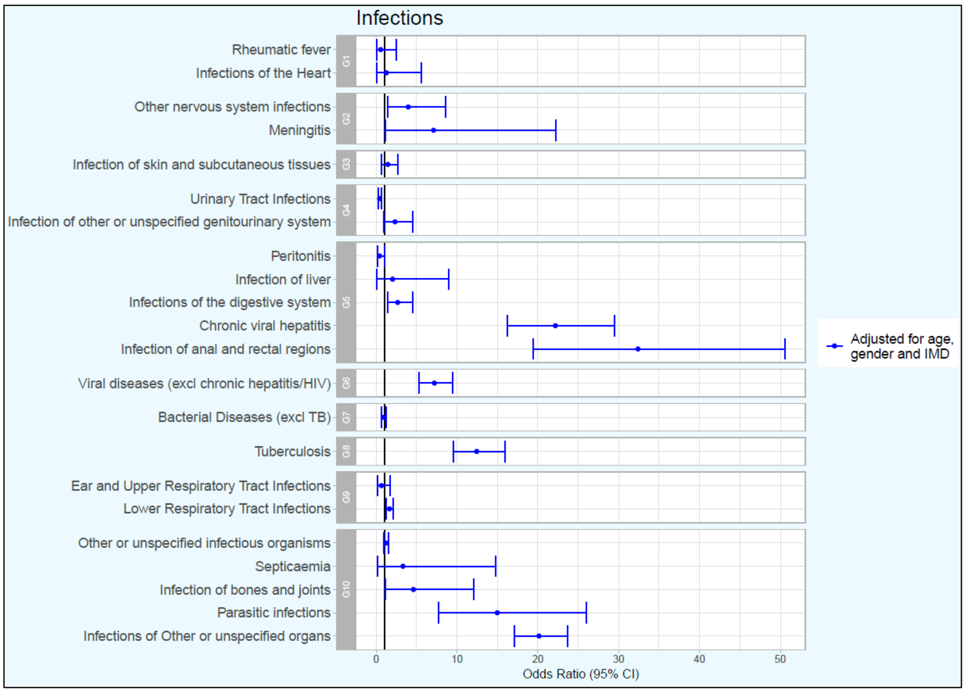

B.

Figure A10. Prevalence and odds ratios of individual infections in adults with HIV compared to those without HIV.

HIV=human immunodeficiency virus. 95%CI=95% confidence interval. OR=odds ratio. IMD = index of multiple deprivation. TB=tuberculosis. CNS=central nervous system. CVS=cardiovascular. GI=gastrointestinal. GU=genitourinary.

G1 = Infection_CVS, G2 = Infection_CNS, G3 = Infection_Skin, G4 = Infection_GU, G5 = Infection_GI, G6 = Infection_Mycoses, G7 = Infection_Viral, G8 = Infection_Bacterial, G9 = Infection_TB, G10 = Infection_Respiratory, G11 = Infection_Other.

Table A13. Prevalence and odds ratios of individual liver disorders in adults with HIV compared to those without HIV.

Health condition	People with HIV, n (%) (N=964)	People without HIV, n (%) (N=941113)	Unadjusted OR (95% CI)	Adjusted for age and sex OR (95% CI)	Adjusted for age, sex and IMD OR (95% CI)
**Liver disorder**					
Alcoholic liver disease	<5	1766 (0.2)	1.66 (0.41-4.32)	1.64 (0.41-4.26)	1.41 (0.35-3.68)
Fatty Liver	11 (1.1)	8169 (0.9)	1.32 (0.68-2.27)	1.39 (0.72-2.39)	1.28 (0.66-2.20)
Liver failure	<5	759 (0.1)	2.58 (0.43-7.99)	2.59 (0.43-8.03)	2.12 (0.35-6.57)
Liver fibrosis, sclerosis and cirrhosis	15 (1.6)	4124 (0.4)	3.59 (2.06-5.76)	3.75 (2.15-6.03)	3.23 (1.85-5.20)
Oesophageal varices	<5	1067 (0.1)	2.75 (0.68-7.16)	2.84 (0.71-7.41)	2.51 (0.62-6.56)
Portal hypertension	<5	1866 (0.2)	2.62 (0.94-5.67)	2.69 (0.96-5.81)	2.32 (0.83-5.01)
